# Efficient Screening for Ternary Molecular Ionic Cocrystals Using a Complementary Mechanosynthesis and Computational Structure Prediction Approach

**DOI:** 10.1002/chem.201904672

**Published:** 2020-03-24

**Authors:** Abeer F. Shunnar, Bhausaheb Dhokale, Durga Prasad Karothu, David H. Bowskill, Isaac J. Sugden, Hector H. Hernandez, Panče Naumov, Sharmarke Mohamed

**Affiliations:** ^1^ Department of Chemistry Khalifa University of Science and Technology P.O. Box 127788 Abu Dhabi UAE; ^2^ New York University Abu Dhabi P.O. Box 129188 Abu Dhabi UAE; ^3^ Molecular Systems Engineering Group Centre for Process Systems Engineering Department of Chemical Engineering Imperial College London London SW7 2AZ UK; ^4^ Department of Biomedical Engineering Center for Membrane and Advanced Water Technology Khalifa University of Science and Technology Masdar Campus P.O. Box 127788 Abu Dhabi UAE

**Keywords:** crystal engineering, crystal structure prediction, green chemistry, mechanosynthesis, molecular ionic cocrystals, X-ray diffraction

## Abstract

The discovery of molecular ionic cocrystals (ICCs) of active pharmaceutical ingredients (APIs) widens the opportunities for optimizing the physicochemical properties of APIs whilst facilitating the delivery of multiple therapeutic agents. However, ICCs are often observed serendipitously in crystallization screens and the factors dictating their crystallization are poorly understood. We demonstrate here that mechanochemical ball milling is a versatile technique for the reproducible synthesis of ternary molecular ICCs in less than 30 min of grinding with or without solvent. Computational crystal structure prediction (CSP) calculations have been performed on ternary molecular ICCs for the first time and the observed crystal structures of all the ICCs were correctly predicted. Periodic dispersion‐corrected DFT calculations revealed that all the ICCs are thermodynamically stable (mean stabilization energy=−2 kJ mol^−1^) relative to the crystallization of a physical mixture of the binary salt and acid. The results suggest that a combined mechanosynthesis and CSP approach could be used to target the synthesis of higher‐order molecular ICCs with functional properties.

## Graphical abstract

**Spot on predictions**! The mechanosynthesis of ternary molecular ionic cocrystals (ICCs) with significantly different physicochemical properties has been achieved in less than 30 min of grinding. The crystal structures of the ICCs were successfully predicted by using computational methods. The results pave the way for the efficient screening of higher‐order multicomponent crystal forms with functional properties (see figure).

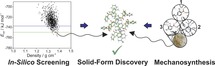

## Introduction

The ability to select the optimal crystal form of an active pharmaceutical ingredient (API) has important economic, regulatory, and clinical consequences.^1^ The past three decades has seen significant progress in our ability to synthesize complex solid forms^2^ by using the principles of crystal engineering,^3^ often aided by computational insight.^4^ Despite this, it remains impossible to be certain that all crystal forms have been discovered in a solid form screen because there are always more variables to investigate than time and resources allow.^5^ Nevertheless, the solid form screen is considered a success if it culminates in the discovery of one or more crystal forms with desirable properties (e.g., solubility, stability, or bioavailability). Within the class of multicomponent crystal forms, binary cocrystals comprising two molecular components have received significant attention^6^ over the past two decades. However, recent reports of the advantages^7^ offered by ionic cocrystals (ICCs) derived from the cocrystallization of APIs with atomic inorganic salts (e.g., NaCl and CaCl_2_) suggest that the range of practically accessible solid forms for optimizing the properties of APIs continues to widen.^8^ This is in the context of recent reports of the synthesis of a quaternary cocrystal^9^ and the first reported synthesis of a six‐component molecular solid.^10^

Here, we focus on the systematic crystallization of ternary molecular ICCs that conform to the empirical formula A^−^ **⋅**BH^+^ **⋅** CH , in which A^−^ and BH^+^ are molecular ions derived from acid (AH)/base (B) proton transfer and CH is an acidic molecular coformer.

We define molecular ICCs as solid forms that 1) are derived from the cocrystallization of organic molecular species (e.g., AH, B, and CH) that are all solids under room temperature and pressure conditions and 2) are sustained by charge‐assisted hydrogen‐bonding interactions that arise as a consequence of one or more proton transfer events. These requirements provide a contrast with ICCs derived from organic molecules and atomic inorganic salts (i.e., the so‐called “organic–inorganic ICCs”)^11^ because such systems do not arise from proton transfer events and display structures that are largely distinguished on the basis of the coordination patterns observed around a metal ion. The above framework also allows us to draw a sharp distinction between molecular ICCs and solvates,^12^ which, unlike ICCs, are derived following the inclusion of one or more volatile liquids. Solvent molecules can often be removed from nonstoichiometric solvates without affecting the structural integrity of the resulting desolvate. By contrast, molecular ICCs are defined by structures featuring three or more molecular species that are all integral to the crystal packing and display a single sharp melting point. Because it is possible to cocrystallize a molecular salt (A^−^ **⋅**BH^+^) with a variety of acidic coformers (CH ), we classify ternary molecular ICCs into two categories based on the chemical structures of AH and CH: Conjugate acid/base ICCs (denoted “CAB‐ICCs”)^13^ and nonconjugate acid/base ICCs (denoted “NCAB‐ICCs”). CAB‐ICCs are derived from experiments in which AH=CH , such that $A^{-}$ and CH are conjugate acid/base pairs. By contrast, NCAB‐ICCs are derived from experiments in which AH≠CH , such that $A^{-}$ and CH are not conjugate acid/base pairs. NCAB‐ICCs potentially offer more scope^14^ for tuning the solid‐state properties of ionizable APIs due to the unrestricted number of AH/CH pairs that can be used to target such solid forms.

**Scheme 1:**
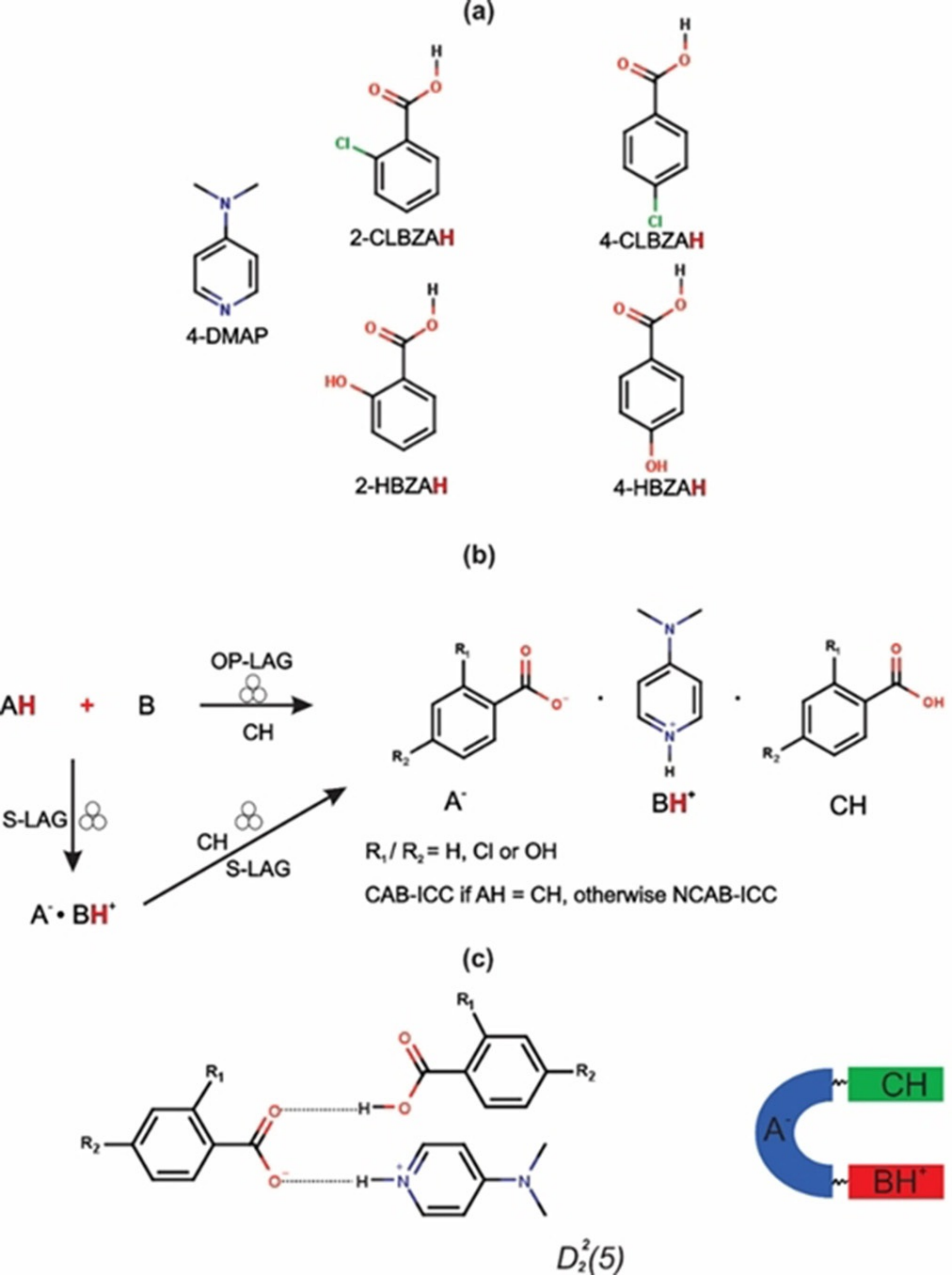
(a) Molecular structures of the base (B=4‐DMAP) and acid (AH) coformers used to target ternary molecular ICCs with the empirical formula A^−^BH^+^CH. AH=2‐CLBZAH, 4‐CLBZAH, 2‐HBZAH, or 4‐HBZAH. The species CH is one of the four acid coformers shown. (b) Reaction schemes for the one‐pot (OP‐LAG) and serial (S‐LAG) liquid‐assisted grinding procedures for the synthesis of ternary molecular ICCs. (c) Illustration of the common $D_{2}^{2}\left(5\right)$ hydrogen‐bonded heterosynthon observed in most of the ICCs studied in this work.

Mechanosynthesis is increasingly being recognized as an efficient and environmentally friendly methodology for a range of chemical transformations.^15^ Recent efforts that aimed to synthesize ICCs7a, 7c, 7e, 7f focused on the cocrystallization of APIs with metal halides, and there have been some documented successes in the mechanosynthesis of such organic–inorganic ICCs.^16^ However, the mechanosynthesis of ternary molecular ICCs remains an underexplored area of research. Here, we demonstrate the potential for using mechanochemical ball milling (under neat grinding (NG) or liquid‐assisted grinding (LAG) conditions)^17^ for the facile screening of CAB‐ICCs and NCAB‐ICCs. The LAG mechanosynthesis of ICCs has been explored by using a serial two‐step liquid‐assisted grinding (S‐LAG) or a one‐pot liquid‐assisted grinding (OP‐LAG) synthetic protocol (Scheme 1). The S‐LAG approach proceeds by the mechanosynthesis of the 1:1 binary salt complex followed by a subsequent step involving stoichiometric amounts of this salt and an acid coformer. By contrast, the OP‐LAG approach proceeds by the direct cocrystallization of the ICC by using a 2:1 (AH:B, CAB‐ICC) or 1:1:1 (AH:CH:B, NCAB‐ICC) stoichiometric ratio of the reactants. Initially, the mechanosynthesis of CAB‐ICCs and NCAB‐ICCs reported exclusively from solution crystallization screens^14^, ^18^ were targeted as a validation of our proposed mechanochemical rapid‐screening protocol. This was followed by mechanosynthesis experiments targeting a novel CAB‐ICC of 2‐chlorobenzoic acid and 4‐dimethylaminopyridine using a coformer replacement strategy based on molecular size‐matching. We compare the thermal stabilities of the ICCs as we screen various combinations of AH/CH pairs. We also assess the role that solvent plays in driving the self‐assembly of molecular ICCs during the mechanosynthesis experiments. Thus, CAB‐ICCs and NCAB‐ICCs have been synthesized (Scheme 1) by the cocrystallization of 4‐dimethylaminopyridine (4‐DMAP) with 2‐chlorobenzoic acid (2‐CLBZAH), 4‐chlorobenzoic acid (4‐CLBZAH), 2‐hydroxybenzoic acid (2‐HBZAH), or 4‐hydroxybenzoic acid (4‐HBZAH).

Despite the proven value of computational crystal structure prediction (CSP) methods in facilitating the discovery of previously unknown solid forms,4c, 19 molecular solids comprising three or more chemical entities are not routinely studied in such work. This reflects the demands on computing resources and the complexity of the search space, which scales exponentially^20^ with the degrees of freedom. Indeed, the chloride salt hydrate (target XXIV) proposed in the sixth blind test^21^ of CSP had the lowest number of attempted predictions by participants of the blind test and only one group was able to generate the experimental structure in their list of predicted structures. Here, we report on the CSP of ternary molecular ICCs for the first time. Initially, we test the success of the computational model in reproducing the crystal structures of known CAB‐ICCs and NCAB‐ICCs displaying rigid molecular conformations. This is followed by a more extensive blind^21^ CSP study on two conformationally flexible systems: A binary salt and a ternary CAB‐ICC derived from 2‐CLBZAH and 4‐DMAP. We use the predicted crystal form landscapes (CFLs) to assess the preferred packing modes of ternary molecular ICCs and the degree to which CSP methods can guide the selection of ICCs in crystallization screens. We also test the hypothesis that the crystallization of ternary molecular ICCs is under thermodynamic control,^22^ and that the use of dispersion‐corrected density functional theory (DFT‐D) energies is diagnostic enough to guide the experimental discovery of ternary molecular ICCs.

## Results and Discussion

### Mechanosynthesis and the solid‐state properties of ICCs

**Figure 1:**
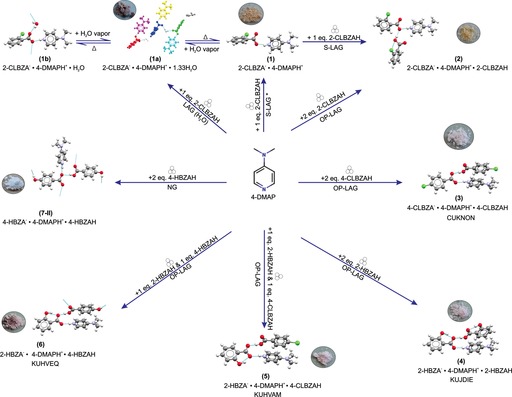
Chemical composition, labeling system, and significant hydrogen‐bonding interactions (colored cyan) for all the solid forms synthesized in this work. The atoms are colored by element for all the solid forms except for **1 a**, for which the species in the asymmetric unit is colored according to symmetry equivalence. Images of the bulk powder sample obtained following the mechanosynthesis of each target solid form is shown where applicable. CSD^23^ reference codes for the ICCs (**3**–**6**) previously reported^14^, ^18^ following solution crystallization screens are indicated in capital letters. Asterisks (*) indicate that the solvent is either MeOH or *i*PrOH

**Figure 2:**
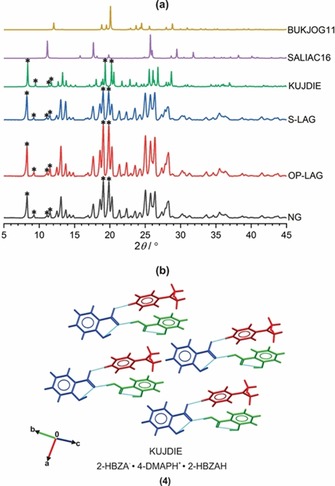
(a) Experimental room‐temperature PXRD patterns for the NG, OP‐LAG (MeOH) and S‐LAG (MeOH) products in experiments targeting CAB‐ICC **4** (Refcode: KUJDIE). The PXRD patterns for 4‐DMAP (Refcode: BUKJOG11), 2‐HBZAH (Refcode: SALIAC16), and KUJDIE are simulated by using the reported single‐crystal structural data. Diagnostic Bragg reflections for the targeted CAB‐ICC are indicated by asterisks (*). (b) Crystal packing for **4** indicating the $D_{2}^{2}\left(5\right)$ synthon between the anion (blue), cation (red), and acid (green) in the ICC structure.

Solution crystallization of ternary molecular ICCs is complicated by the differences in the solubilities of the reactants, which if significant can lead to undesired products or a physical mixture of the starting reactants. This is reflected in the serendipitous solution crystallization of ternary molecular ICCs in experiments targeting the binary salt using a 1:1 stoichiometric ratio of the acid and base.^24^ By contrast, the mechanosynthesis (Scheme 1) of ICCs is attractive in minimizing (OP‐LAG) or completely removing (NG) the role of the solvent during the synthesis of the ICC. We set out to test the potential for the rapid mechanochemical screening of ICCs by using 4‐DMAP and the set of acid coformers shown in Scheme 1. The diversity of solid forms obtained from our mechanosynthesis screens are illustrated in Figure 1. A salt anhydrate (**1**), two salt hydrates (**1 a**, **1 b**), and a CAB‐ICC (**2**) were discovered in mechanosynthesis experiments involving 2‐CLBZAH and 4‐DMAP. The ICCs **3**–**6** (Figure 1) previously reported^14^, ^18^ from solution crystallization screens were all shown to be amenable to mechanosynthesis (see Figures S1–S4 in the Supporting Information). OP‐LAG experiments targeting the CAB‐ICC with the composition 4‐HBZA^−^ **⋅**4‐DMAPH^+^ **⋅**4‐HBZAH (Cambridge Structural Database (CSD) Refcode: CUKNUT; Form I, hereafter **7**‐**I**) did not yield a solid form matching the reported^14^ CUKNUT structure. Instead, a polymorph of this CAB‐ICC (Form II, hereafter **7**‐**II**) was isolated under NG conditions. The mechanosynthesis of ICCs was shown to be feasible with or without the addition of a polar solvent (MeOH, *i*PrOH, or H_2_O, using a solvent/solute ratio (*η*)^25^ of approximately 0.1 μL mg^−1^ for OP‐LAG experiments). ICCs were also shown to be amenable to mechanosynthesis by the serial (S‐LAG) or one‐pot (OP‐LAG) liquid‐assisted grinding route (Scheme 1). Figure 2 illustrates the success in the mechanosynthesis of CAB‐ICC **4** (CSD Refcode: KUJDIE),^18^ generating the same target solid form under NG, S‐LAG, or OP‐LAG conditions. In most cases, the products obtained from NG or OP‐LAG experiments were the same. However, the effect of solvent was found to be significant for experiments involving 4‐HBZAH. For experiments involving a 2:1 stoichiometric ratio of 4‐HBZAH and 4‐DMAP, the NG product was characterized to be Form II of the reported CUKNUT structure (**7**‐**II**), whereas the OP‐LAG (MeOH solvent) product did not match the CUKNUT structure and presented challenges in cell indexing due to the poor crystallinity of the sample. This apparent failure in the mechanosynthesis of **7**‐**I** using MeOH is set in the context of this being the reported^14^ solvent of choice for growing single crystals of **7**‐**I**. In most cases, OP‐LAG experiments using a protic solvent led to detectable improvements in the crystallinity and phase purity of the ICC as monitored by powder XRD (PXRD) measurements. For all the target ICCs (**2**–**7**), the solid form could be synthesized in as little as 15 min of grinding, but favorable conversion from reactants to the product was detected at 30 min of grinding by using the OP‐LAG approach. All ICCs appeared to be stable to amorphization, even after grinding for 60 min.

Molecular ICCs display supramolecular features typically associated with salts as they comprise formally charged ions ($A^{-}$ and ${BH}^{+}$ ) that engage in charge‐assisted hydrogen‐bonding interactions. However, ICCs are also a type of cocrystal because they are derived from the cocrystallization of a binary salt ($A^{-}{BH}^{+}$ ) with another species (e.g., CH). These observations suggest the need for a nuanced perspective when attempting to classify ICCs as their properties do not conform to the seemingly binary choice between a salt or a cocrystal.6c, 26 Some attempts have already been made to classify^27^ multicomponent solid forms in the context of the wider set of possible crystal forms. However, variations in classification continue to exist with some reports describing ICCs as “salt cocrystals”,^28^ “cocrystal salts”,27b or even “acid solvates”.^29^ Despite the lack of a widely accepted classification scheme for such solid forms, what is clear is that the successful synthesis of ternary or higher‐order ICCs offers opportunities^11^ for optimizing the solid‐state properties of ionizable or non‐ionizable molecules. All the ICCs shown in Figure 1 (**2**–**7**) are derived from acid/base pairs with $\Delta {pK}_{a}$ values (Table 1) in the range 4.40–5.99. For NCAB‐ICCs **5** and **6**, the more acidic component (2‐HBZAH) ionizes in the process of assembling the ternary ICC, in agreement with chemical intuition. However, in the context of the “salt–cocrystal continuum”,^30^ ICCs defy the predictive power of existing empirical rules (i.e., the “$\Delta {pK}_{a}$ rule”) for targeting neutral or charged complexes of molecules because they comprise neutral and ionized forms of the same molecule(s).^31^

**Table 1:** Properties of the ICCs obtained following mechanosynthesis.

ICC	ICC composition	ICC type	ICC hydrogen‐bond graph set	${\Delta pK}_{a}$ ^[a]^	$\Delta V_{vdW}$ ^[a]^ [Å^3^]	$\Delta C_{k}$ ^[b]^ [%]	Melting point onset [°C]	Melting point peak [°C]	Predicted energetic rank on CFL^[c]^	$\Delta E_{ICC}\;[kJ\;{mol}^{-1}$ ]^[d]^
**2**	2‐CLBZA^−^ · 4‐DMAPH^+^ · 2‐CLBZAH	CAB	$D_{1}^{1}\left(2\right)$	5.71	5.14 (2.41)	0	91.85	94.96	5	−0.11
**3**	4‐CLBZA^−^ · 4‐DMAPH^+^ · 4‐CLBZAH	CAB	$D_{2}^{2}\left(5\right)$	4.71	5.33 (2.46)	−0.2	152.09	156.43	1	−1.45
**4**	2‐HBZA^−^ · 4‐DMAPH^+^ · 2‐HBZAH	CAB	$D_{2}^{2}\left(5\right)$	5.99	10.68 (2.41)	0.6	108.00	110.47	6	−0.69
**5**	2‐HBZA^−^ · 4‐DMAPH^+^ · 4‐CLBZAH	NCAB	$D_{2}^{2}\left(5\right)$	5.99	10.68 (7.81)	1.2	148.10	150.72	2	−0.63
**6**	2‐HBZA^−^ · 4‐DMAPH^+^ · 4‐HBZAH	NCAB	$D_{2}^{2}\left(5\right)$	5.99	10.68 (2.33)	1.1	147.78	151.21	21	−4.86
**7‐II**	4‐HBZA^−^ · 4‐DMAPH^+^ · 4‐HBZAH	CAB	$D_{1}^{1}\left(2\right)$	4.40	10.76 (2.41)	2.8	162.49	164.77	–	−4.42

[a] These properties were calculated by using the calculator plug‐ins in MarvinSketch.^32^ The $\Delta V_{vdW}$ column lists the absolute difference in the calculated^32^ van der Waals volumes for the cation and anion (and in parentheses for the acid and anion) in each ICC. [b] The Kitaigorodsky packing coefficients ($C_{k}$ ) were calculated by using PLATON:^33^ $\Delta C_{k}=C_{k}\left(ICC\right)-C_{k}\left(salt\right)$ . For each ICC, the salt crystal structures were retrieved from the CSD by searching for structures comprising the molecular ions found in the ICC (see Table S3 in the Supporting Information for CSD refcodes). [c] The energetic rank indicates the relative stability of the experimental ICC on the computed crystal form landscape (CFL). The lowest possible rank is 1, which corresponds to the global minimum in lattice energy. The energetic ranking for **7‐II** could not be computed because the newly determined Form II structure (**7**‐**II**) was outside the scope of the rigid‐body searches for hypothetical crystal structures. [d] The ICC stabilization energy ($\Delta E_{ICC}$ ) relates to the DFT‐D estimate of the lattice energy of each ICC relative to the energies of the binary salt and acid [see Eq. (1) and the accompanying discussion in the main text].

**Figure 3:**
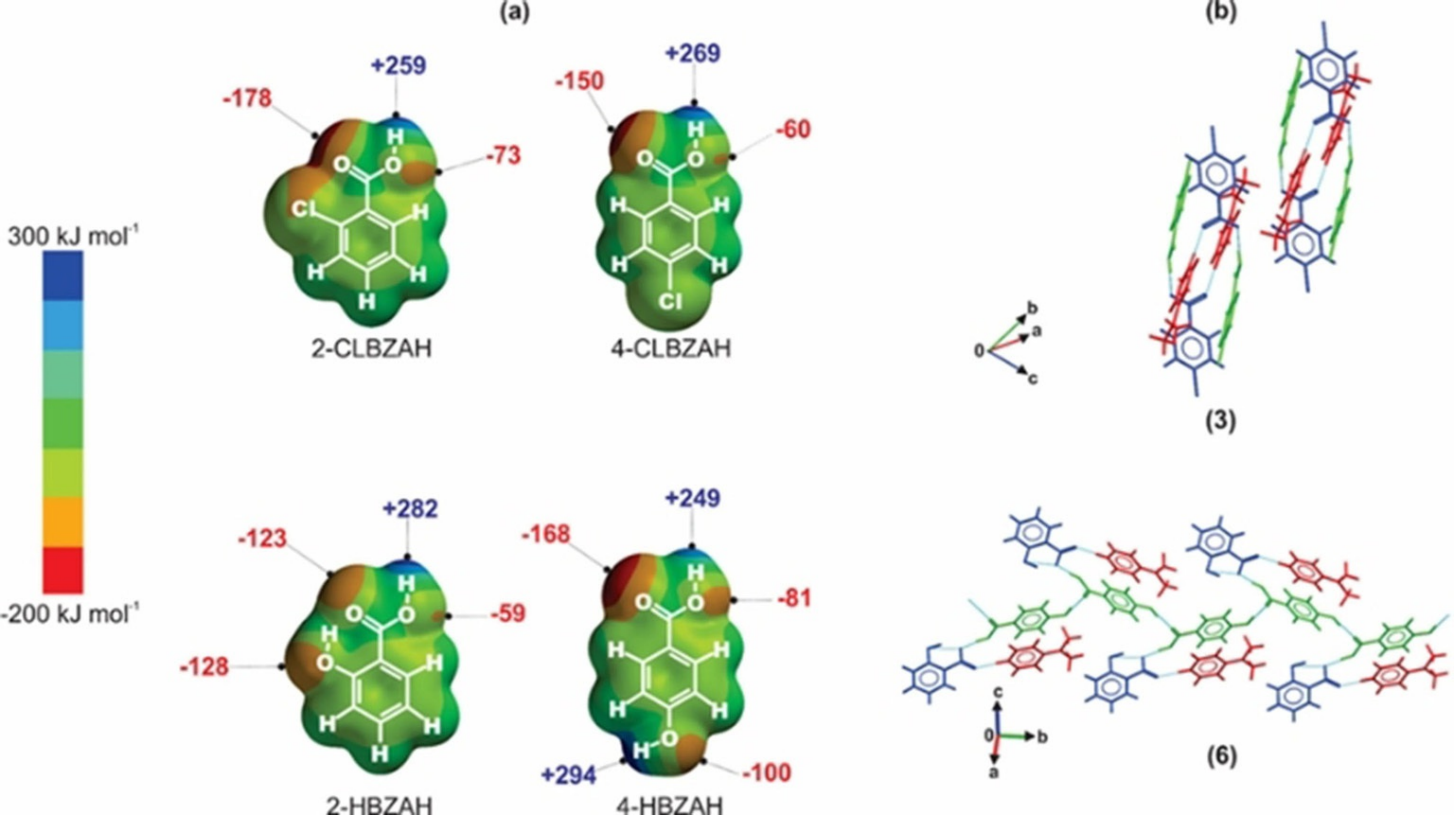
(a) Molecular electrostatic potential (MEP) surfaces for 2‐CLBZAH, 4‐CLBZAH, 2‐HBZAH, and 4‐HBZAH (potential values in kJ mol^−1^). Only the potentials for hydrogen‐bond donor (blue) and acceptor (red) groups capable of engaging in intermolecular hydrogen bonding are indicated. (b) Comparison of the hydrogen‐bonding interactions (colored cyan) in CAB‐ICC **3** (4‐CLBZA^−^ **⋅**4‐DMAPH^+^ **⋅**4‐CLBZAH) with those observed in NCAB‐ICC **6** (2‐HBZA^−^ **⋅**4‐DMAPH^+^ **⋅**4‐HBZAH). In the packing diagrams for both ICCs the anion (blue), cation (red), and acid (green) are colored according to symmetry equivalence.

Although supramolecular synthons^3^ are valuable tools for understanding the most significant intermolecular interactions driving the assembly of binary cocrystals,^34^ the complicated interplay of competing weak intermolecular interactions in higher‐order crystals makes it difficult to predict the dominant intermolecular interactions in such systems. For cocrystals displaying competing hydrogen‐bond donor and acceptor sites in the constituent molecules, Hunter proposed^35^ a method for estimating the likelihood of cocrystallization on the basis of the differences in the calculated interaction site pairing energies for the cocrystal and the constituent molecules. Such interaction site pairing energies can be derived from the molecular electrostatic potentials (MEPs) of the molecules that make up the cocrystal. This approach has been used to successfully predict not only whether a cocrystal could form,35b but also the dominant supramolecular synthons in cocrystals.^36^ For all ICCs in our series (**2**–**7**), the R_1_COO^−^⋅⋅⋅H−^+^NR_2_ hydrogen bond is observed between the molecular ions, and this is expected given the strong coulombic forces that stabilize this donor–acceptor pairing. The MEPs for the acid coformers (Figure 3) used in our mechanosynthesis screens were calculated (Figure 3) in an attempt to quantify the relative strengths of the remaining set of possible hydrogen‐bond donor/acceptor pairings. For all ICCs in our series, the carboxylic acid OH donor of the coformer engages in discrete hydrogen‐bonding interactions with one of the carboxylate oxygen atoms of the anion. This is expected for ICCs such as **3** (Figure 3) in which the acid coformer only bears one hydrogen‐bond donor (Figure 3). However, for ICCs such as **6** that comprise the 4‐HBZAH coformer, the acid now possesses two donor sites capable of engaging in intermolecular hydrogen bonding with the molecular ions. The calculated MEP surface for 4‐HBZAH (Figure 3) shows that the carboxylic acid OH donor has a potential energy that is approximately 45 kJ mol^−1^ less than that of the *para*‐substituted OH donor, which implies that the latter is a better hydrogen‐bond donor. However, the observed hydrogen‐bonding interactions in **6** (Figure 3) show that the carboxylic acid OH donor interacts with the carboxylate oxygen acceptor. This suggests that although the principle of the strongest hydrogen‐bond donor interacting with the strongest hydrogen‐bond acceptor may be sufficient in predicting binary cocrystal formation,35b the dominant role of electrostatics in stabilizing ICCs means that MEP surfaces derived in vacuo are not adequate for predicting all the observed supramolecular synthons in ICCs.

The physical appearances (Figure 1) and melting points (Table 1) of the ICCs were found to be sensitive to the chemical identities of the AH /CH pairs used to crystallize the ICC. All ICCs displayed sharp melting endotherms (see Figure S6 in the Supporting Information) with onsets (Table 1) ranging from 91.85 (**2**) to 162.49 °C (**7**‐**II**). Comparison of the van der Waals volumes ($\Delta V_{vdW}$ ) of the cation/anion (${BH}^{+}$ /$A^{-}$ ) and coformer/anion (CH /$A^{-}$ ) pairs in the ICCs (Table 1) shows negligible differences (2 Å^3^<Δ*V*_vdW_<11 Å^3^) in the intrinsic molecular volumes of all three hydrogen‐bonded species in the series **2**–**7**. The small sample size considered here prevents us from drawing general conclusions about the significance of this observation. However, the consistency in the differences in the intrinsic molecular volumes across the series (Table 1) is worthy of further investigation as it may point towards the necessary conditions for favorable coformer exchange leading to NCAB‐ICCs such as **5** and **6**.

Comparison of the Kitaigorodsky packing coefficients ($C_{k}$ )^37^ of the molecular ICCs in our series with the packing coefficients of their respective molecular salts as reported in the CSD (see Table S3 in the Supporting Information for refcodes) show that only **5**, **6**, and **7**‐**II** display >1 % improvement (Table 1) in $C_{k}$ upon switching from the salt to the ICC. The finding that **2**–**4** show little or no change (Table 1) in the packing efficiency of the species in the crystal as we switch from the binary salt to the ternary ICC is consistent with previous observations^22^ that poor crystal packing of salt ions is not a satisfactory explanation for rationalizing ICC formation. CSP studies (rigid molecular conformations) on the set of ICCs with previously reported crystal structures (**3**–**6** and **7**‐**I**) have shown (see Tables S6–S10) that all experimentally determined solid forms could be predicted within an energy range of 20 kJ mol^−1^ with respect to the global minimum (GM) structure. The newly characterized **7**‐**II** polymorph was outside the scope of the rigid‐body CSP search for **7** due to the molecular flexibility exhibited by **7**‐**II**. However, the Form I CUKNUT polymorph (**7**‐**I**) was predicted at rank 28 during the search for hypothetical rigid‐body ICC structures. The relatively high energy for **7**‐**I** is consistent with DFT‐D estimates of the relative energies for the two polymorphs (see below), which show that **7**‐**II** is approximately 2.56 kJ mol^−1^ more stable than **7**‐**I**. Overall, the finding that most of the ICCs (**2**–**5**) display CFLs for which the experimental crystal structure is amongst the 10 most stable structures and the fact that all previously characterized ICC structures (**3**–**6** and **7**‐**I**) could be predicted, suggest that computational CSP methods can play an important role in the discovery of ternary molecular ICCs.

### Experimental crystal structures

**Figure 4:**
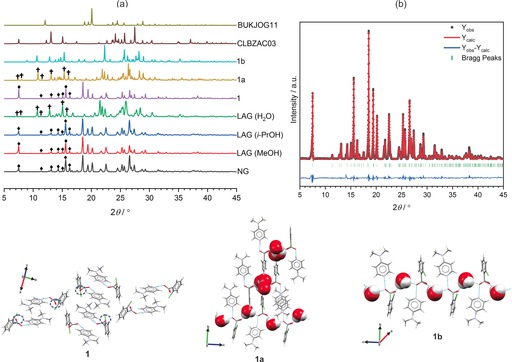
(a) PXRD patterns following NG and LAG (MeOH, *i*PrOH, or H_2_O) mechanosynthesis experiments involving a 1:1 stoichiometric ratio of 2‐CLBZAH and 4‐DMAP. PXRD patterns for **1**, **1 a**, **1 b**, CLBZAC03 (2‐CLBZAH), and BUKJOG11 (4‐DMAP) are simulated by using the single‐crystal structural data. Diagnostic Bragg reflections for facilitating comparison of the PXRD patterns obtained following the NG or LAG (MeOH or *i*PrOH) experiments with the PXRD pattern simulated by using the single‐crystal structural data of **1** are indicated by diamonds (♦), whereas the reflections for comparing the PXRD pattern of the LAG (H_2_O) product with the pattern simulated by using the single‐crystal structural data of **1 a** are indicated by daggers (†). (b) Final Rietveld refinement fit for the binary salt 2‐CLBZA^−^ **⋅**4‐DMAPH^+^ (**1**). (c) Crystal packing diagrams for **1**, **1 a**, and **1 b** illustrating the role that water plays in satisfying the unused hydrogen‐bond acceptors in **1** (indicated in blue‐dotted circles).

Prior to our work, there were no reported binary or ternary multicomponent solid forms derived from the 2‐CLBZAH/4‐DMAP acid/base pair. The 2‐CLBZA^−^ **⋅**4‐DMAPH^+^ (**1**) binary salt was successfully synthesized in quantitative yield under NG and LAG (MeOH or *i*PrOH) conditions (Figure 4 a) by using a 1:1 stoichiometric ratio of the acid/base pair. Attempts to grow diffraction‐quality single crystals of the mechanosynthesis product of the salt **1** by using a range of solvents failed, with the exception of experiments involving *i*PrOH, which led to hydrated crystals containing 1.33 (**1 a**) or 1 (**1 b**) water molecule(s) per mole of the ion pair (see Table S1 in the Supporting Information). The LAG mechanosynthesis of **1** using water led to a sample whose PXRD pattern matched (Figure 4 a) that simulated from the single‐crystal structure of **1 a**. However, LAG using the same *i*PrOH solvent used to grow single crystals **1 a** and **1 b** led instead to the salt anhydrate **1**, which suggests that the milling product is sensitive to the water activity in the crystallization medium. Heating a powder sample of **1 a** to a temperature of 110 °C led to facile dehydration to furnish **1** (see Figure S5). The facile hydration of **1** under solution crystallization conditions meant that diffraction‐quality single crystals of the salt anhydrate could not be grown. Instead, the crystal structure of **1** (see Table S2) was solved from the PXRD data by using the Monte Carlo simulated annealing method^38^ with the final Rietveld^39^ fit (Figure 4 b) converging at an $R_{wp}$ of 5.72 %. It is clear from the experimental crystal structure of **1** (Figure 4 c) that each symmetry related anion displays a single unused hydrogen‐bond acceptor. Comparing **1** with the corresponding hydrate crystal structures shows that this “frustration” in hydrogen bonding is resolved (Figure 4 c) by the inclusion of water, leading to solvent molecules bridging isolated units of hydrogen‐bonded molecular ions in **1 a** and **1 b**.

**Figure 5:**
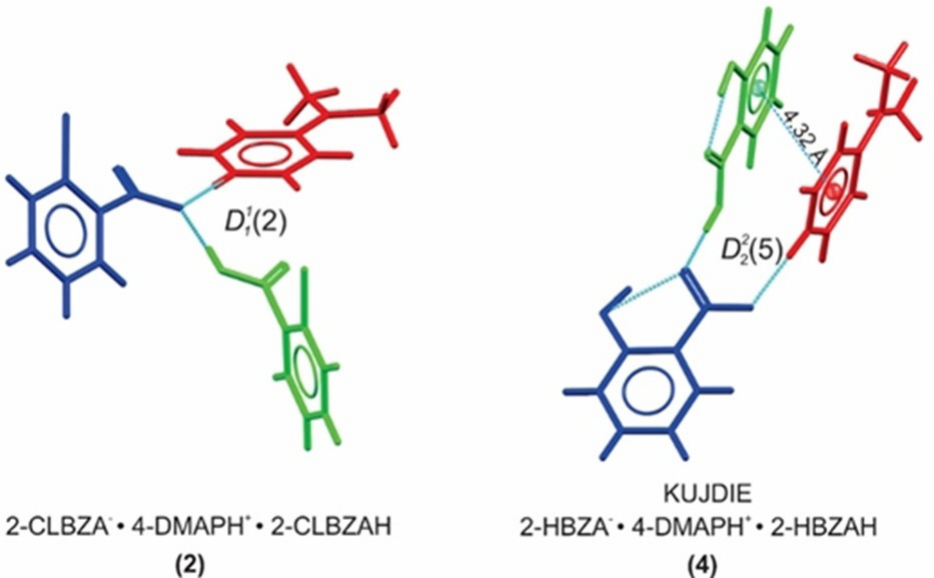
Observed hydrogen‐bonding (colored cyan) motifs in CAB‐ICCs **2** and **4**. Despite the comparable molecular volumes of 2‐HBZAH and 2‐CLBZAH, significant differences in the crystal packing and solid‐state properties (Table 1) of **2** and **4** are observed. The centroid–centroid distance (4.32 Å) in the π–π stacking interactions between the acid and cation of **4** are indicated. Anions are colored in blue, cations in red, and acid coformers in green.

The comparable van der Waals volumes of 2‐HBZAH and 2‐CLBZAH (Table 1) coupled with the existence of a reported^18^ CAB‐ICC (**4**, KUJDIE) with composition 2‐HBZA^−^ **⋅**4‐DMAPH^+^ **⋅**2‐HBZAH allowed us to target the synthesis of the 2‐CLBZA^−^ **⋅**4‐DMAPH^+^ **⋅**2‐CLBZAH CAB‐ICC (**2**) by using a coformer replacement strategy based on molecular size‐matching. Suitable colorless crystals of **2** with block morphology were grown by using *i*PrOH solvent and the crystal structure of the ICC (see Table S1) was determined by using single‐crystal X‐ray diffraction (SXRD) methods. Comparison of the supramolecular synthons of **2** and **4** (Figure 5) show, however, that **2** adopts a $D_{1}^{1}\left(2\right)$ synthon whereas **4** adopts the common $D_{2}^{2}\left(5\right)$ synthon that is observed for most of the ICCs (**3**–**6**) in our series. Inspection of the molecular conformations adopted by the $A^{-}/CH$ pairs in the ICCs that display the $D_{2}^{2}\left(5\right)$ synthon (**3**–**6**) shows that they are all approximately planar. The planar molecular conformations in these ICCs facilitates close molecular association marked by π–π interactions between the acid and cation with centroid–centroid distances (Figure 5) ranging from 3.60 to 4.32 Å. By contrast, the torsions defining the rotation of the carboxylic acid and carboxylate groups in **2** are −71 and −48°, respectively, leading to significant deviations from planarity (Figure 5) and by extension the absence of the close molecular association that appears to be the hallmark of those ICCs displaying the $D_{2}^{2}\left(5\right)$ synthon. Thus, although a molecular‐size matching approach may be successful in targeting CAB‐ICCs that differ in the identities of the $A^{-}/CH$ pairs, these observations illustrate the challenges in attempting to extrapolate the dominant supramolecular synthons of ICCs on the basis of the synthons observed in structurally related systems.

**Figure 6:**
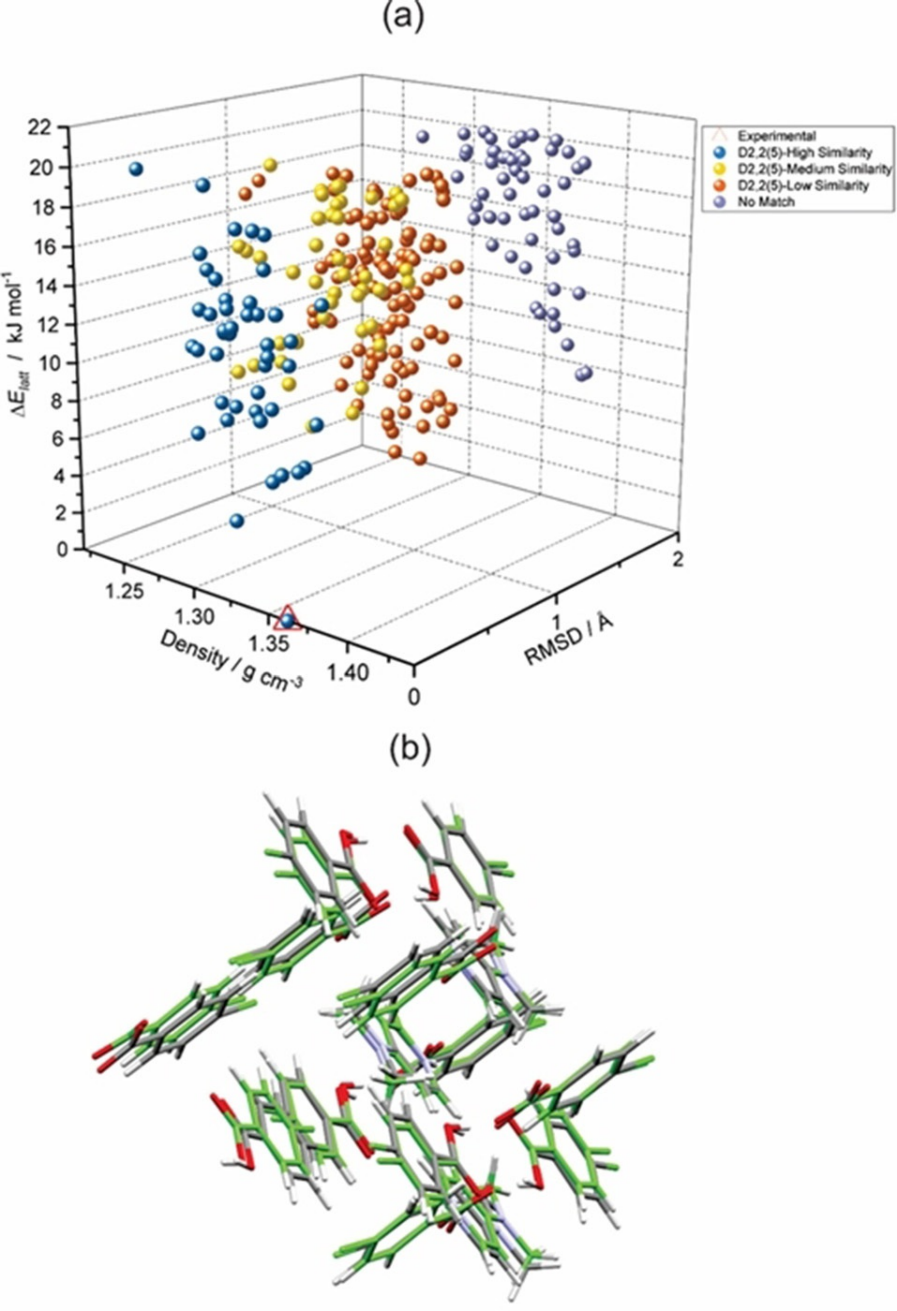
(a) Crystal form landscape (CFL) for CAB‐ICC **3** (CUKNON) illustrating the diversity of possible packing polymorphs classified in terms of the RMSD ( Å) of the predicted hydrogen‐bond motif for each polymorph relative to that of the experimental GM structure. $\Delta E_{latt}$ is the energy of each polymorph relative to the GM structure. The hydrogen bond similarities were judged according to the following cut‐offs: High: 0 ≤ RMSD ≤ 0.3; medium: 0.3 < RMSD ≤ 0.8; low: RMSD > 0.8. (b) Overlay of the crystal‐packing structures of the predicted GM structure (colored by element) and the experimental CAB‐ICC **3** structure (colored green) after matching 15 molecules in the coordination spheres of the two structures.

The CFL of 4‐CLBZA^−^ **⋅**4‐DMAPH^+^ **⋅**4‐CLBZAH (**3**, Refcode: CUKNON)^14^ was analyzed (Figure 6) to gain a deeper understanding of the alternative packing preferences of the molecular components in ICCs displaying the $D_{2}^{2}\left(5\right)$ synthon. The experimental CUKNON structure of **3** is the GM in lattice energy and is approximately 3.4 kJ mol^−1^ more stable than the rank 2 structure on the CFL. The top three ranked ICC structures all display the same $D_{2}^{2}\left(5\right)$ synthon. Comparing the hydrogen‐bond geometry of each predicted polymorph of **3** with the geometry of the reference GM structure revealed that 31 % of all structures within 10 kJ mol^−1^ of the GM adopt the $D_{2}^{2}\left(5\right)$ synthon with a high degree of similarity (root‐mean‐square deviation (RMSD)=0–0.3 Å). Within the usual caveats^40^ of static lattice energy calculations, the computational model therefore suggests that the reported^14^ experimental structure of **3** is energetically favored and there appears to be an energetic driving force for the nucleation of crystals that display the $D_{2}^{2}\left(5\right)$ synthon. Because most of the predicted low‐energy polymorphs belong to the same cluster of structures displaying the $D_{2}^{2}\left(5\right)$ synthon, it follows that the absence of energetically competitive structures with alternative packing arrangements to CUKNON implies a minimal risk of packing polymorphism in this ICC system.

**Figure 7:**
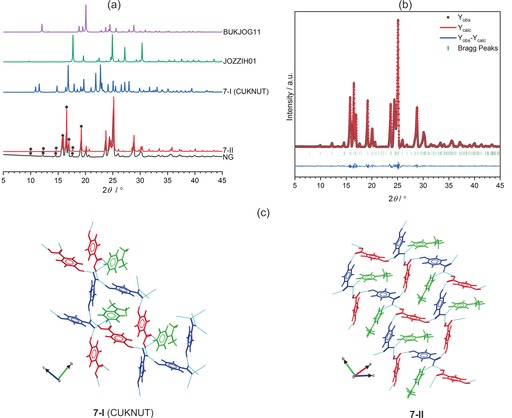
(a) Comparison of the simulated PXRD patterns from the single‐crystal structural data of **7**‐**I** (CUKNUT), **7**‐**II**, 4‐HBZAH (JOZZIH01), and 4‐DMAP (BUKJOG11) with the experimental PXRD pattern obtained following neat grinding (NG) of a 2:1 molar ratio of 4‐HBZAH and 4‐DMAP. Asterisks (*) indicate diagnostic Bragg reflections for comparing the experimental PXRD pattern of the NG product with the simulated PXRD pattern of **7**‐**II**. (b) Final Rietveld refinement fit for Form II of 4‐dimethylaminopyridinium**⋅**4‐hydroxybenzoate**⋅**4‐hydroxybenzoic acid (**7**‐**II**). (c) Comparison of the hydrogen‐bond motifs in **7**‐**I** (CUKNUT) and **7**‐**II**. Anions are colored in blue, cations in green, and acid coformers in red.

NG experiments targeting the CAB‐ICC **7**‐**I** (CUKNUT)^14^ led instead to the characterization of a new polymorph (**7**‐**II**) of this ICC (Figure 7 a). The crystal structure (see Table S2 in the Supporting Information) for **7**‐**II** was solved from the PXRD data of the NG product by using the Monte Carlo simulated annealing^38^ method (Figure 7 b, final Rietveld^39^ fit yielded $R_{wp}$ =3.60 %). The **7**‐**II** polymorph crystallizes in the same ${P2}_{1}$ space group observed for **7**‐**I**. Variable‐cell DFT‐D geometry optimizations confirmed **7**‐**II** to be a true lattice energy minimum with sensible hydrogen‐bond geometries. The DFT‐D optimizations were used to support the identification of the acidic proton positions in **7**‐**II** given the low scattering factor of the hydrogen atom. In both polymorphs, all hydrogen‐bond donors and acceptors are satisfied (Figure 7 c) and the structures display discrete $D_{1}^{1}\left(2\right)$ synthons between the acid/anion and cation/anion pairs. In addition to these discrete interactions, **7**‐**II** displays a $C_{2}^{2}\left(16\right)$ chain motif between the acid and the anion (Figure 7 c) with one cation trapped inside each chain. The packing coefficient ($C_{k})$ of **7**‐**II** is approximately 2.8 % higher (Table 1) than that observed for the 4‐HBZA^−^ **⋅**4‐DMAPH^+^ binary salt (CSD Refcode: SOLGUX),^41^ whereas the $C_{k}$ of **7**‐**I** is identical to that of the salt. The **7**‐**II** polymorph displays the highest onset of melting (162.49 °C) of all the ICCs in the series **2**–**7** (Table 1). Challenges in the mechanosynthesis of pure **7**‐**I** prevented us from characterizing its physical properties relative to **7**‐**II**.

### Blind structure prediction and periodic DFT‐D estimates of the energetic driving force for ICC formation

**Figure 8:**
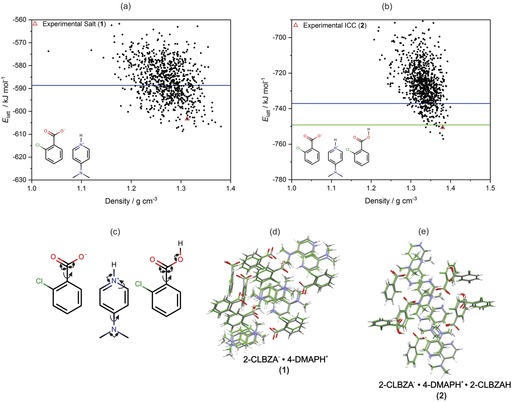
Predicted crystal form landscapes (CFLs) for (a) the binary salt **1** and (b) CAB‐ICC **2**. Each black circle represents a hypothetical polymorph on the lattice energy landscape. The experimental structures of **1** and **2** are indicated by red triangles. The blue line in each CFL indicates an energy range of 20 kJ mol^−1^ relative to the predicted GM structure. The green line in (b) indicates the sum of the lattice energies for the GM structures in the CFLs of the acid and salt. (c) Molecular degrees of freedom for each component of **1** and **2** that were optimized during the final ranking of the crystal lattice energies by using CrystalOptimizer.^70^ Curly arrows indicate torsions and square arrows indicate angles. Structural overlays between the experimental and matching predicted crystal structures are shown for (d) the binary salt **1** and (e) CAB‐ICC **2**.

The CFLs for the 2‐CLBZA^−^ **⋅**4‐DMAPH^+^ binary salt (**1**) and the 2‐CLBZA^−^ **⋅**4‐DMAPH^+^ **⋅**2‐CLBZAH ternary CAB‐ICC (**2**) were calculated (Figure 8) from first principles under blind test^21^ conditions. Both systems display molecular flexibility and reflect challenging targets for current methods of CSP. The blind nature of the CSP work allowed us to test the predictive value of the computed CFLs for guiding the selection of ternary molecular ICCs. The binary salt structure of **1** was predicted to be the 19th most stable polymorph in the CFL (Figure 8 a) and lies approximately 5.4 kJ mol^−1^ above the GM structure. The successful prediction of the crystal structure for **1** supports the experimentally determined structure and highlights the value of CSP methods for aiding structure solution from PXRD data. Notably, the CFL for **1** contains many low‐energy/high‐density polymorphs within 20 kJ mol^−1^ that are more stable and efficiently packed than the experimental structure of **1** (Figure 8 a). The facile hydration of **1** to form a nonstoichiometric hydrate (**1 a**) and the observation of a dense CFL with many more stable polymorphs of the anhydrate therefore suggest that there is scope for the discovery of more stable anhydrous polymorphs of **1**. By contrast, the crystal structure of CAB‐ICC **2** was successfully predicted to be the 5th most stable polymorph, with a relative energy of 6.5 kJ mol^−1^ with respect to the GM structure. The CFL (Figure 8 b) for this system is by comparison much less dense in the low‐energy region with fewer stable lattice energy minima. Notably, CAB‐ICC **2** is predicted to have a CFL with a range of polymorphs (including the experimental structure) that are more stable than the sum of the lattice energies for the most stable salt and acid polymorphs (i.e., below the green line in Figure 8 b), which indicates a thermodynamic preference for ICC formation. The successful prediction of the experimental structure of this flexible ternary ICC system under blind test conditions suggests that CSP methods are now at a stage of maturity that they can be used to quantify the risks of polymorphism and aid the discovery of higher‐order multicomponent crystal forms.

**Figure 9:**
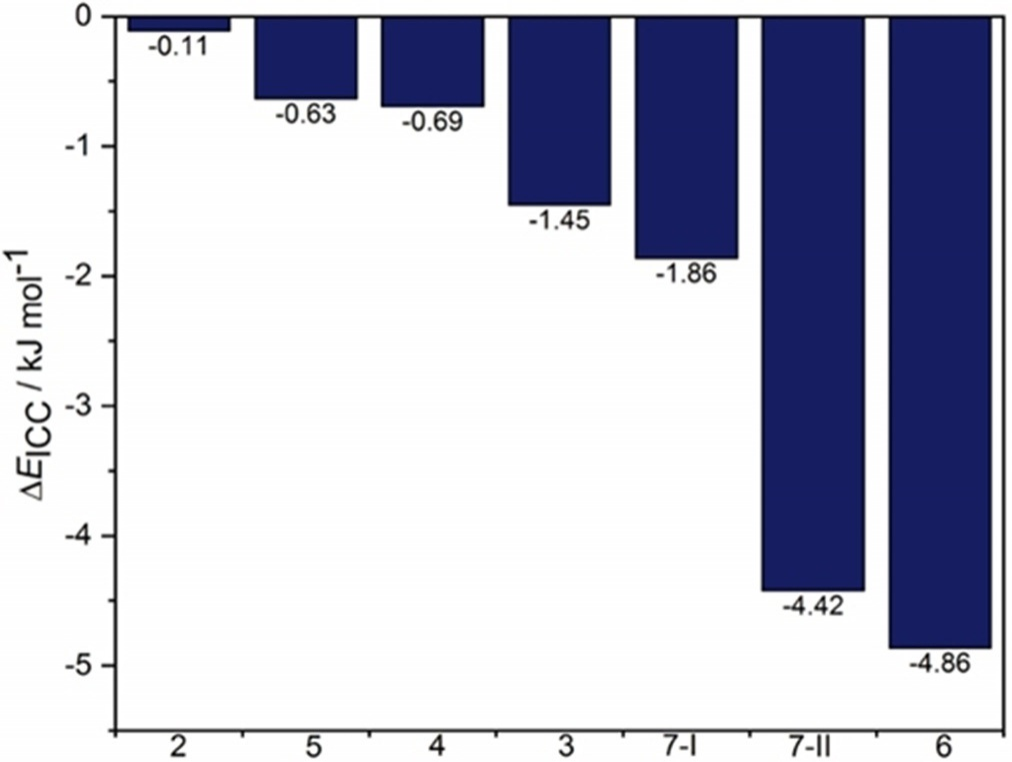
Estimate of the energetic driving force ($\Delta E_{ICC})$ for crystallizing each of the ternary molecular ICCs (**2**–**7**) in our study relative to the competing process of crystallizing a physical mixture of the binary salt and acid. We assume crystallization is under thermodynamic control and that negative values for $\Delta E_{ICC}$ imply favorable ICC formation. **7**‐**I** refers to the Form I CUKNUT structure whereas **7**‐**II** is the Form II structure for this CAB‐ICC determined in this work.

In an attempt to obtain accurate lattice energies for probing the thermodynamic driving force for ICC formation, the experimental crystal structures of all ICCs in our series (**2**–**7**) were subjected to variable‐cell DFT‐D geometry optimizations by using the TPSS meta‐GGA functional^42^ with D3 dispersion correction.^43^ Similar optimizations were performed for the corresponding salt and acid components used to construct the ICC (see Table S3 in the Supporting Information). The energetic driving force for crystallizing an ICC (${\Delta E}_{ICC}$ ) as compared with the competing process of crystallizing a physical mixture of the binary salt and acid was estimated according to Equation (1):

(1) $$\Delta E_{ICC}=\frac{E_{ICC}}{Z_{ICC}}-\left(\frac{2}{3}\frac{E_{SALT}}{Z_{SALT}}+\frac{1}{3}\frac{E_{ACID}}{Z_{ACID}}\right)$$

in which $E_{i}$ are the electronic structure energies of the unit cells with the energies normalized by the number of molecules in the unit cell (Z ) with the comparison made between a mole of ICC and 2/3 and 1/3 of a mole of salt and acid, respectively. For all ICCs in our series (**2**–**7**), we find that ${\Delta E}_{ICC}$ is negative, which suggests that crystallization of the ICC is energetically favored (Figure 9). The mean value of ${\Delta E}_{ICC}$ is −2 kJ mol^−1^ across the series. However, the ICC is energetically favored by less than 1 kJ mol^−1^ for **2**, **4**, and **5**, which suggests that the use of DFT‐D relative energies alone would not have been sufficient to rule out the formation of a physical mixture of the binary salt and acid. For **3**, **6**, and **7**, ${\Delta E}_{ICC}$ ranges from −1.45 to −4.86 kJ mol^−1^. Although the thermodynamic driving force for the crystallization of ICCs has yet to be studied in a rigorous manner, several attempts have been made to quantify the thermodynamic driving force for the crystallization of binary cocrystals. Issa et al.^44^ studied 26 cocrystals of 4‐aminobenzoic acid, succinic acid, and caffeine by using a multipole electrostatic model combined with an empirical potential for describing the dispersion–repulsion interactions. In general, they found that the experimentally observed cocrystals tended to be more stable than the pure components. However, several exceptions to this rule were observed, such as the finding that an experimentally observed urea**⋅**succinic acid cocrystal was calculated to be 12.09 kJ mol^−1^ higher in energy relative to its pure components. A more recent study by Taylor and Day^45^ employed DFT‐D calculations using the PBE functional and D3 dispersion correction to evaluate the relative stabilities of 350 cocrystals. From this larger study it was concluded that 95 % of experimentally observed binary cocrystals are thermodynamically stable relative to their pure component energies. The average stabilizing energy of these systems was found to be −8 kJ mol^−1^. This is in contrast to our findings for the thermodynamic stabilization of the ternary molecular ICCs in our limited set, which appear to be in better agreement with the energy differences of pure component polymorphs.40b, 46

## Conclusion

A rational approach for the discovery of multicomponent crystal forms is critical to the formulation of active pharmaceutical ingredients with optimal solid‐state properties. For ternary molecular ICCs, the differences in the solubilities of the reactants can be significant and this can limit the success of solution crystallization screens. Our work has shown that ternary molecular ICCs of the type $A^{-}\cdot {BH}^{+}\cdot CH$ can be synthesized by mechanochemical ball milling in as little as 30 min. For most of the systems surveyed, NG conditions are sufficient to target the ternary ICC, although LAG experiments did lead to improved phase purity and crystallinity for most systems. ICCs display thermal stabilities that can be tuned depending on the chemical identities of AH and CH . The binary salt complex of 4‐dimethylaminopyridinium**⋅**2‐chlorobenzoate (**1**) proved challenging to crystallize under solution crystallization conditions given the relatively high affinity of this salt for water, which led to hydrated crystals containing 1.33 (**1 a**) and 1 (**1 b**) water molecule(s) per ion pair. The salt complex of **1** was synthesized in quantitative yield under NG or LAG conditions by using the ball‐milling technique and the novel ternary CAB‐ICC 4‐dimethylaminopyridinium**⋅**2‐chlorobenzoate**⋅**2‐chlorobenzoic acid (**2**) was synthesized by using a coformer replacement strategy based on molecular‐size matching. All the ICCs with rigid molecular conformations prepared in this study (**3**–**6** and **7**‐**I**) were shown to have crystal packings that could be predicted from first principles by using computational CSP methods. The crystal structures for the conformationally flexible binary salt complex **1** and the ternary CAB‐ICC **2** determined in this work were also successfully predicted under blind test conditions using no prior information other than the input molecular structures. A Form II structure for 4‐dimethylaminopyridinium**⋅**4‐hydroxybenzoate**⋅**4‐hydroxybenzoic acid (**7**‐**II**) was synthesized in NG experiments by using a 2:1 molar ratio of the acid and base. This polymorph was shown to have the highest thermal stability of all the ICCs surveyed and its discovery illustrates the importance of mechanochemical screening for higher‐order cocrystals using NG conditions as the absence of solvent can provide access to solid forms not seen in solution. DFT‐D calculations on all the ICCs surveyed showed good agreement with experimental observations in predicting that all ICC systems are thermodynamically stable relative to the crystallization of a physical mixture of the salt and acid. However, the mean stabilization energy of ICCs in our limited series was found to be −2 kJ mol^−1^, which is lower than the −8 kJ mol^−1^ previously reported for binary cocrystals using a comparable DFT‐D model. Overall, the successful computational prediction and experimental realization of previously unknown ternary molecular ICCs suggest that a complementary mechanosynthesis and crystal structure prediction approach could aid the rapid screening and selection of functional molecular ICCs with improved physicochemical properties.

## Experimental Section

**Mechanosynthesis of ICCs**: The chemicals 4‐DMAP, 4‐CLBZAH, 2‐HBZAH, and 4‐HBZAH were purchased from Sigma–Aldrich (≥98 % GC), whereas 2‐CLBZAH was purchased from Acros Organics (≥98 % GC). All chemicals were used in experiments as supplied. The CAB‐ICCs were synthesized by using 2:1 acid/base molar ratios whereas experiments targeting NCAB‐ICCs employed 1:1:1 molar ratios of acid_1_/acid_2_/base. OP‐LAG experiments were performed by using MeOH, *i*PrOH, or distilled water using a solvent/solute ratio (*η*)^25^ of approximately 0.1 μL mg^−1^. For S‐LAG experiments, solvent (30 μL ) was added to a stoichiometric ratio of the reactants in each step. Unless otherwise stated, assume that the solvent of choice in S‐LAG or OP‐LAG experiments was MeOH. For all acid‐base pairs, the calculated^32^ aqueous $\Delta {pK}_{a}$ was in the range 4.40–5.99, which indicates that there is a high probability^26^, ^30^, ^47^ of ionization to form charged molecular ions during the mechanosynthesis experiments. Mechanosynthesis experiments were performed by using a Retsch MM200 mixer mill equipped with 10 mL capacity stainless‐steel grinding jars and two 7 mm diameter stainless‐steel grinding balls per jar. All milling experiments were performed at a frequency of 25 Hz. To test the reproducibility of the mechanosynthesis protocol as well as the stability of the ICC towards amorphization, the grinding experiments were repeated for each reaction over successively longer time periods spanning 15, 30, or 60 min. PXRD data were collected on the milling products obtained after 60 min of grinding.

**Single‐crystal X‐ray diffraction**: The OP‐LAG (*i*PrOH solvent) products obtained after milling a 1:1 or 1:2 molar ratio of 4‐DMAP**/**2‐CLBZAH were used to grow single crystals of the salt hydrates (**1 a** and **1 b**) and CAB‐ICC (**2**), respectively. Diffraction‐quality single crystals of **1 a/1 b** and **2** were grown by the slow evaporation of *i*PrOH using 0.718 or 0.689 mmol of the ground reactants dissolved in *i*PrOH (5 mL). The *i*PrOH solvent was allowed to evaporate slowly in a ventilated fume hood over a period of 7 days. The experiment targeting the binary salt complex led to a mixture containing single crystals of **1 a** and **1 b**. Single‐crystal X‐ray diffraction data for **1 a**, **1 b**, and **2** were collected by using a Bruker Duo three‐circle diffractometer equipped with a Cobra cooling device (Oxford Cryosystems) with graphite‐monochromated Mo_Kα_ (λ=0.71073 Å) radiation and a Photon 100 CMOS area detector. The optimum strategy for data collection involved different sets of ϕ and ω scans with $0.5^{\circ }$ steps in ϕ/ω . Data collection, integration, scaling, and absorption corrections were performed by using Bruker Apex 3 software.^48^ Data reduction was performed by using SAINT^49^ and XPREP.^50^ All the data were corrected for Lorentzian, polarization, and absorption effects by using the SADABS^51^ program. The structures were solved by using SHELXT‐2014/5^52^ and refined by full‐matrix least‐squares methods based on $F^{2}$ against all reflections using SHELXL‐2014/7.^53^ The Olex2 graphical user interface^54^ was used to visualize and manipulate the structure solution and refinement process. Two water molecules in **1 a** exhibited disorder, which was modelled and treated by using a set of constraints, namely EADP and EXYZ. All non‐hydrogen atom positions were located by using difference Fourier methods and refined anisotropically. The positions of the hydrogen atoms bonded to carbon were set by using the HFIX command, whereas the positions of all the other hydrogen atoms were located in the difference Fourier map and freely refined. Publication‐quality images were generated by using Mercury 4.0.0,^55^ X‐Seed,^56^ and POV‐Ray.^57^ A summary of the crystallographic data for **1 a**, **1 b**, and **2** can be found in Table S1 in the Supporting Information.

CCDC https://www.ccdc.cam.ac.uk/services/structures?id=doi:10.1002/chem.201904672 (**1 a**, **1 b**, **2**, **7‐II** and **1**) contain the supplementary crystallographic data for this paper. These data are provided free of charge by http://www.ccdc.cam.ac.uk/.

**Powder X‐ray diffraction**: PXRD data were collected on a PANalytical Empyrean diffractometer equipped with an X′Celerator RTMS detector. Diffraction experiments were performed in Bragg–Brentano reflection geometry using nickel‐filtered Cu_Kα_ radiation. PXRD data were collected in the 2θ angular range 4–50${}^{\circ }$ , but due to the lack of any significant diffraction peaks at the extremes, the 2θ range 5–45${}^{\circ }$ was used for comparison with the simulated PXRD patterns as well as for the structure solution of **1** and **7‐II**. Cell indexing and systematic absence determinations for **1** (MeOH LAG product) and **7**‐**II** (NG product) were performed by using the TREOR90^58^ and X‐Cell^59^ programs, respectively, as implemented in the Reflex module of BIOVIA Materials Studio 8.0.^60^ The background was fitted by using a six‐order polynomial and the peak profiles were modelled by using a pseudo‐Voigt function. Pawley refinement was performed on the indexed unit cell by using the Reflex module. The starting molecular geometries of **1** and **7**‐**II** were calculated in the gas phase at the M06/6‐31G(d,p) level of theory by using Gaussian 09.^61^ The Forcite module of BIOVIA Materials Studio 8.0^60^ was used to optimize the relative positions of the species in the indexed unit cell by using the Fine (**1**) or Ultra‐fine (**7**‐**II**) quality setting for the convergence criteria of the energy, forces, and displacement. During this initial Forcite geometry optimization, rigid‐body constraints were imposed on the molecular conformations and the unit cell parameters were not allowed to vary. The Dreiding force field was used during the geometry optimization and atomic charges were derived by using the Gasteiger method. The crystal structures for **1** and **7**‐**II** were solved by using the Reflex PowderSolve^38^ module of Materials Studio 8.0, which uses a Monte Carlo simulated annealing technique. In addition to the degrees of freedom (DOFs) defining the positions and relative orientations of the molecular units in the asymmetric unit, additional DOFs for the torsion angles defining the rotation of the NMe_2_, carboxylate, or carboxylic acid groups were also defined prior to initiating the Monte Carlo search. Hydrogen atoms were explicitly modelled during the Monte Carlo search for the global minimum structure on the $R_{wp}$ surface. The number of Monte Carlo steps required to converge on the correct solution was set automatically by Reflex based on the specified DOFs for each system, leading to a total of 24 million and 417 million steps for **1** and **7**‐**II**, respectively. Rietveld^39^ refinement was performed within Reflex and all relevant flexible torsions and bond lengths and angles were refined. The March–Dollase preferred orientation correction was applied. All non‐hydrogen atom temperature factors were refined anisotropically, whereas hydrogen atoms were treated as isotropic. The final Rietveld cycle yielded an $R_{wp}$ of 5.72 % for **1** and 3.60 % for **7**‐**II**. A summary of the crystallographic data for **1** and **7**‐**II** can be found in Table S2 in the Supporting Information.

**Differential scanning calorimetry and thermogravimetric analysis**: Differential scanning calorimetry and thermogravimetric analysis were performed by using NETZSCH STA 449F3 Jupiter and NETZSCH STA 401 F1 Pegasus instruments, respectively. A ceramic crucible was used in both experiments. Samples were purged by a stream of dry nitrogen gas and heated at a rate 5 °C min^−1^ over the temperature range 30–300 °C.

**Computational crystal structure prediction**: Two sets of crystal structure prediction (CSP) data were generated: Set 1 consisted of the predicted crystal form landscapes (CFLs) for known rigid CAB‐ICCs and NCAB‐ICCs **3**–**7** and set 2 consisted of generating the CFLs for the flexible binary salt **1** and CAB‐ICC **2**. For the set 1 data, the assumed molecular conformations were calculated at the M06/6‐31G(d,p) level of theory, whereas for set 2, the calculations were performed at the PBEPBE/6‐311+G(d,p) level of theory. In all cases, molecular conformations were calculated in the gas phase by using Gaussian 09. Searches for hypothetical crystal structures for **3**–**7** assumed the calculated gas‐phase conformational energy minima as input, and these conformations were constrained throughout the simulation. For systems **1** and **2**, flexible torsions were determined through second derivatives and finite difference perturbations. Local approximate models (LAMs) were constructed by using a uniform grid along the one‐dimensional DOFs, at 30° increments. The global search was performed by using CrystalPredictor II,^62^ using the smoothed intramolecular potential,^63^ with 500×10^3^ minimizations for **1** and 1 million for **2**. During the initial structure generation stage, the electrostatic contributions towards the intermolecular forces were estimated by using atomic electrostatic potential charges derived from the ab initio wave functions. Dispersion–repulsion contributions towards the lattice energy were estimated by using a Buckingham exp‐6 function with the potential parameters for C, H_C_ (hydrogen attached to carbon), N, O, and Cl from the work of Williams and co‐workers^64^ as well as the parameters for H_N_ (hydrogen attached to nitrogen)^65^ and H_O_ (hydrogen attached to oxygen),^66^ which were subsequently determined by fitting to crystal structures containing N−H⋅⋅⋅O=C interactions and carboxylic acid structures, respectively. For each search, a final clustering step was performed to remove all duplicate structures with lattice energies within 0.2 kJ mol^−1^, cell volumes within 1.0 Å^3^, and a powder pattern similarity index^55^ of at least 0.97. The Crystal Packing Similarity module of Mercury 4.0.0^55^ was used to match the predicted and experimental crystal structures. This was achieved by estimating the RMSD for matching at least 15 molecules (RMSD_15_) in the coordination spheres of the experimental and predicted structures using a tolerance of 20 % for the distances and 20${}^{\circ }$ for the angles. For set 1, the 1000 most stable structures produced following clustering were passed to DMACRYS^67^ for lattice energy minimization using a distributed multipole model^68^ for the electrostatic contribution towards the lattice energy. Multipoles were calculated up to rank 4 (hexadecapole) for all atoms by performing a distributed multipole analysis of the ab initio charge density using GDMA2.2.^69^ For set 2, CrystalOptimizer^70^ was used to refine the 1000 lowest‐energy structures at the PBEPBE/6‐311+G(d,p) level of theory, with additional flexibility introduced as indicated in Figure 8 c. All lattice energies in the reported CFLs are given per formula unit.

**Molecular electrostatic potentials**: The assumed molecular conformations for computing the molecular electrostatic potentials (MEPs) were those calculated following gas‐phase geometry optimization at the M06/6‐31G(d,p) level of theory by using Gaussian 09. The MEPs for all the acid coformers in Scheme 1 were calculated in vacuo by using the TPSS‐D3 density functional with the 6‐311+G** basis set. Local maxima and minima on the MEP surface (0.002 e a.u.^−1^ isosurface) were determined by using a positive point charge in a vacuum as a probe. The calculations led to the interaction energy (in kJ mol^−1^) between the positive point probe and the surface of the molecule at the point of contact. The MEP surfaces were computed by using Spartan 18.^71^

**Solid‐state periodic DFT‐D geometry optimizations**: All periodic DFT‐D calculations were performed in VASP^72^ which uses the projector‐augmented wave (PAW) 72b, 72c method with plane‐wave basis sets and PAW pseudo‐potentials. The combination of the TPSS meta‐GGA functional^42^ and D3 dispersion correction^43^ was chosen for optimization of the crystal structures due to the good performance of this combination in previous benchmark studies of the X23 test set^73^ as well as its demonstrated ability to correctly determine relative polymorph stabilities.^74^ We made use of a large basis set with an energy cut‐off of 1000 eV and a tight K ‐point mesh with the maximum K ‐point distance set to 2*π* × 0.025 Å^−1^ using a Γ‐centered Monkhorst–Pack scheme. Convergence of each self‐consistent field cycle was set to 10^−6^ eV and the geometry was considered converged when all forces were below 0.01 eV Å^−1^. Starting from the experimentally determined crystal structures, lattice parameters and the fractional coordinates of the atom positions were optimized simultaneously. Careful attention was paid to check that lattice parameters did not change significantly during the optimization to mitigate the extent of Pulay stresses.

## Conflict of interest

The authors declare no conflict of interest.

## Supporting information

As a service to our authors and readers, this journal provides supporting information supplied by the authors. Such materials are peer reviewed and may be re‐organized for online delivery, but are not copy‐edited or typeset. Technical support issues arising from supporting information (other than missing files) should be addressed to the authors.

Supplementary
