# Supplementary material for: Efficient Screening for Ternary Molecular Ionic Cocrystals Using a Complementary Mechanosynthesis and Computational Structure Prediction Approach
Source: Chemistry. 2020 Mar 24;26(21):4752–65. doi: 10.1002/chem.201904672 (PMC7187361; doi:10.1002/chem.201904672)
Supplement: Supplementary file 1 — Supplementary [file CHEM-26-4752-s001.pdf]

Chemistry–A European Journal

Supporting Information

# Efficient Screening for Ternary Molecular Ionic Cocrystals Using a Complementary Mechanosynthesis and Computational Structure Prediction Approach

Abeer F. Shunnar,[a] Bhausaheb Dhokale,[a] Durga Prasad Karothu,[b] David H. Bowskill,[c] Isaac J. Sugden,[c] Hector H. Hernandez,[d] Panče Naumov,[b] and Sharmarke Mohamed*[a]

## Supporting Information (SI)

# Contents


1. Crystallographic tables for experimentally determined structures ........ 3

1.1 Table S1: Single crystal X-ray diffraction data ........ 3

1.2 Table S2: Powder X-ray diffraction data ........ 4

2. PXRD patterns for mechanosynthesis products ........ 5

2.1 Figure S1: 2-chlorobenzoate 4-dimethylaminopyridinium 2-chlorobenzoic acid CAB-ICC (2) ........ 5

2.2 Figure S2: 4-chlorobenzoate 4-dimethylaminopyridinium 4-chlorobenzoic acid CAB-ICC (3; CUKNON) ........ 6

2.3 Figure S3: 2-hydroxybenzoate 4-dimethylaminopyridinium 4-chlorobenzoic acid NCAB-ICC (5; KUHVAM) ........ 7

2.4 Figure S4: 2-hydroxybenzoate 4-dimethylaminopyridinium 4-hydroxybenzoic acid NCAB-ICC (6; KUHVEQ) ........ 8

3. DSC and TGA curves for synthesized solid forms ........ 9

3.1 Figure S5: Combined DSC/TGA curves for 2-$CLBZA^{-}$·4-$DMAPH^{+}$ (1) and 2-$CLBZA^{-}$·4-$DMAPH^{+}$·1.33$H_2O$ (1a) ........ 9

3.3 Figure S6: DSC curves for all ICC solid forms (2-7) ........ 10

4. Computational modelling ........ 11

4.1 Table S3: DFT-D estimates of the lattice energies for ICCs and the corresponding binary salt and acid coformers ........ 11

4.2 Table S4: Selected most stable predicted structures for binary salt 1 ........ 12

4.3 Table S5: Selected most stable predicted structures for CAB-ICC 2 ........ 13

4.4 Table S6: Selected most stable predicted structures for CAB-ICC 3 ........ 14

4.5 Table S7: Selected most stable predicted structures for CAB-ICC 4 ........ 15

4.6 Table S8: Selected most stable predicted structures for NCAB-ICC 5 ........ 16

4.7 Table S9: Selected most stable predicted structures for NCAB-ICC 6 ........ 17

4.8 Table S10: Selected most stable predicted structures for CAB-ICC 7 ........ 18

# 1. Crystallographic tables for experimentally determined structures

## 1.1 Table S1: Single crystal X-ray diffraction data

| Solid Form | 1a | 1b | 2 |
|---|---|---|---|
| Empirical Formula | $C_{42}H_{53}Cl_3N_6O_{9.97}$ | $C_{14}H_{17}ClN_2O_3$ | $C_{21}H_{20}Cl_2N_2O_4$ |
| Formula weight | 907.8 | 296.8 | 435.29 |
| Temperature (K) | 100 | 100 | 100 |
| Crystal system | Monoclinic | Monoclinic | Triclinic |
| Space group | $P2_1/c$ | $P2_1/c$ | $P\bar{1}$ |
| $a$ / Å | 7.1684(5) | 7.0022(6) | 7.2526(10) |
| $b$ / Å | 48.9852(35) | 16.6101(12) | 7.9415(11) |
| $c$ / Å | 12.5940(9) | 12.4391(10) | 17.6801(23) |
| $\alpha$ / º | 90 | 90 | 90.670(3) |
| $\beta$ / º | 101.483(2) | 98.082(3) | 94.352(3) |
| $\gamma$ / º | 90 | 90 | 92.383(3) |
| Volume / Å$^3$ | 4333.8(6) | 1432.39(6) | 1014.39(4) |
| $Z$ | 4 | 4 | 2 |
| Density / (g cm$^{-3}$) | 1.39 | 1.38 | 1.42 |
| $\mu$ / mm$^{-1}$ | 0.276 | 0.275 | 0.351 |
| $F_{000}$ | 1911 | 624 | 452 |
| $h_{min}, h_{max}$ | -8, 8 | -8, 8 | -8, 8 |
| $k_{min}, k_{max}$ | -58, 58 | -20, 20 | -9, 9 |
| $l_{min}, l_{max}$ | -14, 14 | -15, 15 | -21, 21 |
| No. of measured reflections | 42472 | 12031 | 9049 |
| No. of unique reflections | 7596 | 2804 | 3945 |
| No. of reflections used | 7155 | 2585 | 3257 |
| $R_{all}$, $R_{obs}$ | 0.068, 0.064 | 0.040, 0.036 | 0.056, 0.043 |
| $wR_{2,all}$, $wR_{2,obs}$ | 0.141, 0.139 | 0.093, 0.091 | 0.104, 0.100 |
| $\Delta\rho_{min,max}$ / (e Å$^{-3}$) | -0.782, 0.542 | -0.205, 0.534 | -0.357, 0.341 |
| *GooF* | 1.150 | 1.045 | 1.059 |
| CCDC Deposition No. | 1956859 | 1956860 | 1956861 |

Table S1: Crystallographic data for solid forms 1a, 1b and 2 as determined via single crystal X-ray diffraction methods.

## 1.2 Table S2: Powder X-ray diffraction data

| Solid Form | 1 | 7-II |
|---|---|---|
| Empirical Formula | $C_{14}H_{15}N_2ClO_2$ | $C_{21}H_{22}N_2O_6$ |
| Formula weight | 278.7 | 398.4 |
| Crystal system | Monoclinic | Monoclinic |
| Space group | $P2_1/a$ | $P2_1$ |
| $a$ / Å | 10.6958 | 11.1975 |
| $b$ / Å | 23.4783 | 14.3657 |
| $c$ / Å | 7.2615 | 6.0073 |
| $\alpha$ / ° | 90 | 90 |
| $\beta$ / ° | 129.7622 | 91.4054 |
| $\gamma$ / ° | 90 | 90 |
| Volume / Å$^3$ | 1401.74 | 966.043 |
| $Z$ | 4 | 2 |
| $\mathbf{2\theta}$ range | 5-50 | 5-50 |
| Zero point correction | -0.01399 | -0.07311 |
| | | |
| Rietveld refinement results | | |
| $\boldsymbol{R_{wp}}$ | 5.72 | 3.60 |
| $\boldsymbol{R_{wp}}$ (w/o bck) | 5.60 | 3.56 |
| $\boldsymbol{R_p}$ | 9.80 | 5.72 |
| CCDC Deposition No. | 1956863 | 1956862 |

Table S2: Crystallographic data for solid forms 1 and 7-II. Crystal structures were solved using Monte Carlo simulated annealing and refined using the Rietveld refinement method.

## 2. PXRD patterns for mechanosynthesis products

### 2.1 Figure S1: 4-dimethylaminopyridinium 2-chlorobenzoate 2-chlorobenzoic acid CAB-ICC (2)

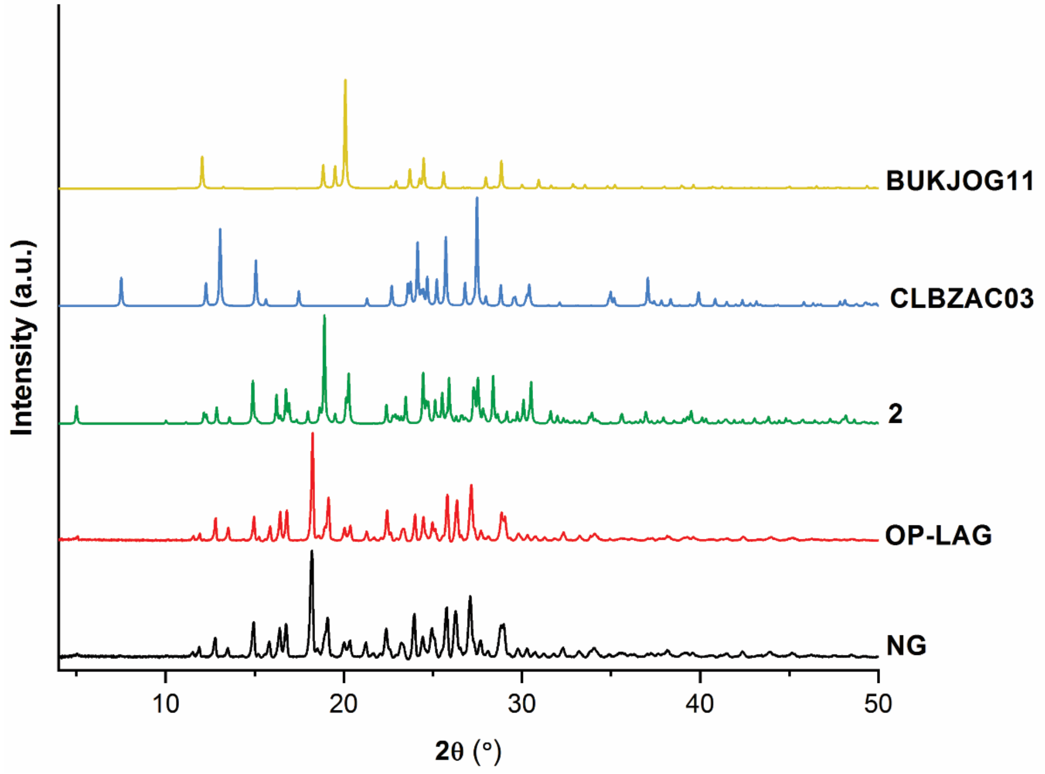


**Figure S1:** Comparison of the experimental PXRD patterns for the neat grinding (NG) and one-pot liquid-assisted grinding (OP-LAG, MeOH) products with the simulated PXRD pattern from the single crystal structure of CAB-ICC **2**. CLBZAC03 and BUKJOG11 are the simulated PXRD patterns from the single crystal structures of 2-chlorobenzoic acid and 4-dimethylaminopyridine respectively.

2.2 Figure S2: 4-dimethylaminopyridinium 4-chlorobenzoate 4-chlorobenzoic acid CAB-ICC (3; CUKNON)

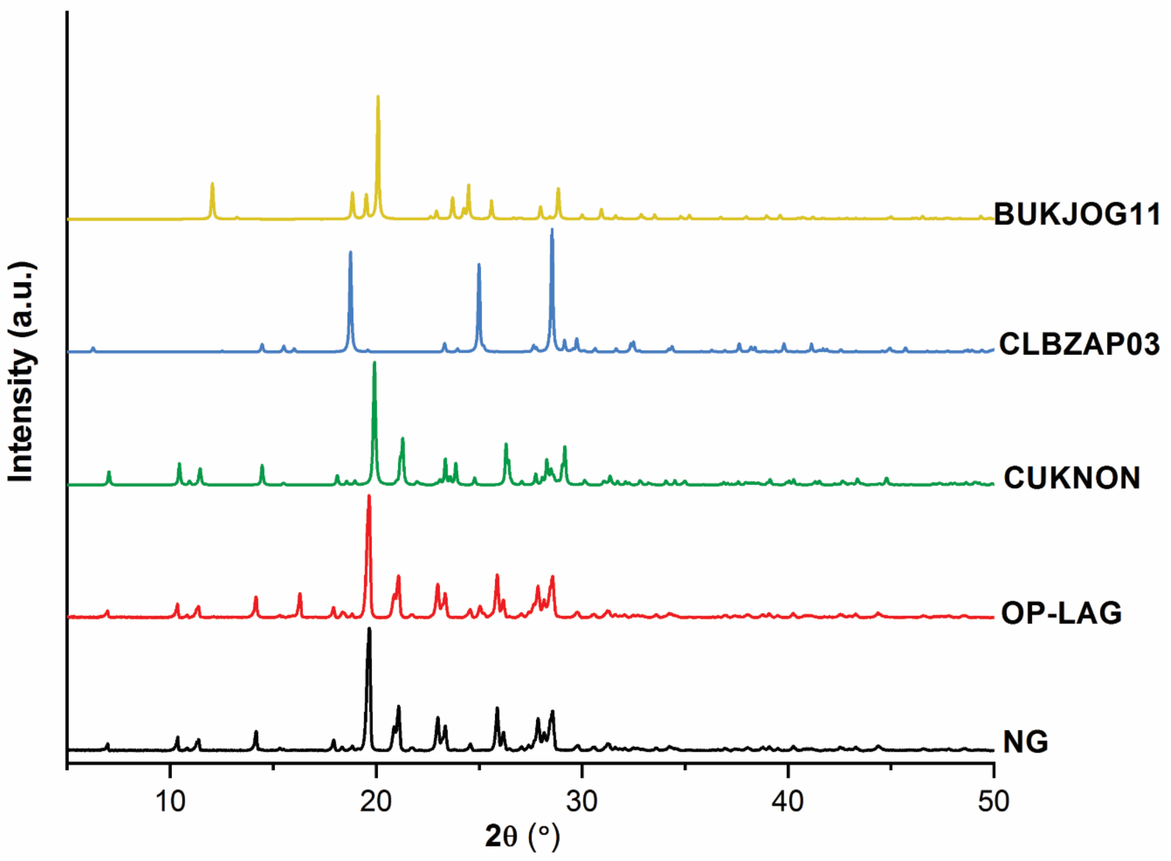


Figure S2: Comparison of the experimental PXRD patterns for the neat grinding (NG) and one-pot liquid-assisted grinding (OP-LAG, MeOH) products with the simulated PXRD pattern from the single crystal structure of CAB-ICC **3** (CSD Refcode: CUKNON). CLBZAP03 and BUKJOG11 are the simulated PXRD patterns from the single crystal structures of 4-chlorobenzoic acid and 4-dimethylaminopyridine respectively.

2.3 Figure S3: 4-dimethylaminopyridinium 2-hydroxybenzoate 4-chlorobenzoic acid NCAB-ICC (5; KUHVAM)

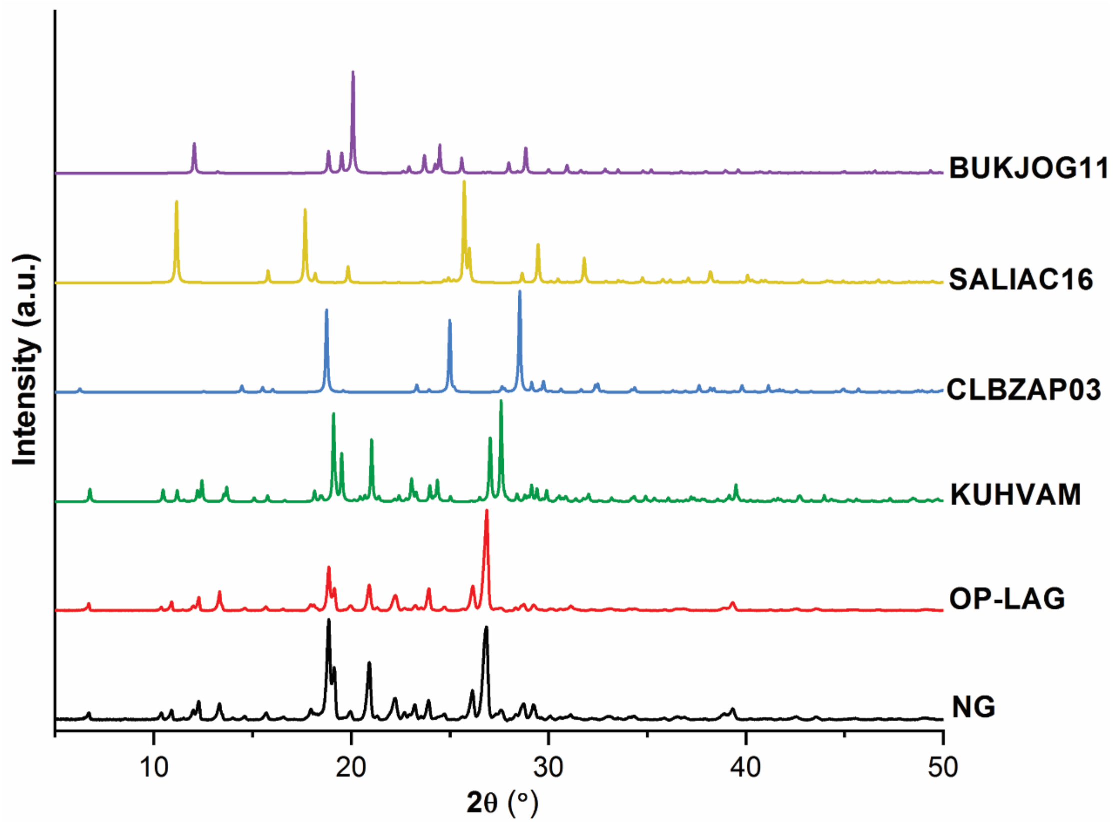


**Figure S3:** Comparison of the experimental PXRD patterns for the neat grinding (NG) and one-pot liquid-assisted grinding (OP-LAG, MeOH) products with the simulated PXRD pattern from the single crystal structure of NCAB-ICC **5** (CSD Refcode: KUHVAM). CLBZAP03, SALIAC16 and BUKJOG11 are the simulated PXRD patterns from the single crystal structures of 4-chlorobenzoic acid, 2-hydroxybenzoic acid and 4-dimethylaminopyridine respectively.

2.4 Figure S4: 4-dimethylaminopyridinium 2-hydroxybenzoate 4-hydroxybenzoic acid NCAB-ICC (6; KUHVEQ)

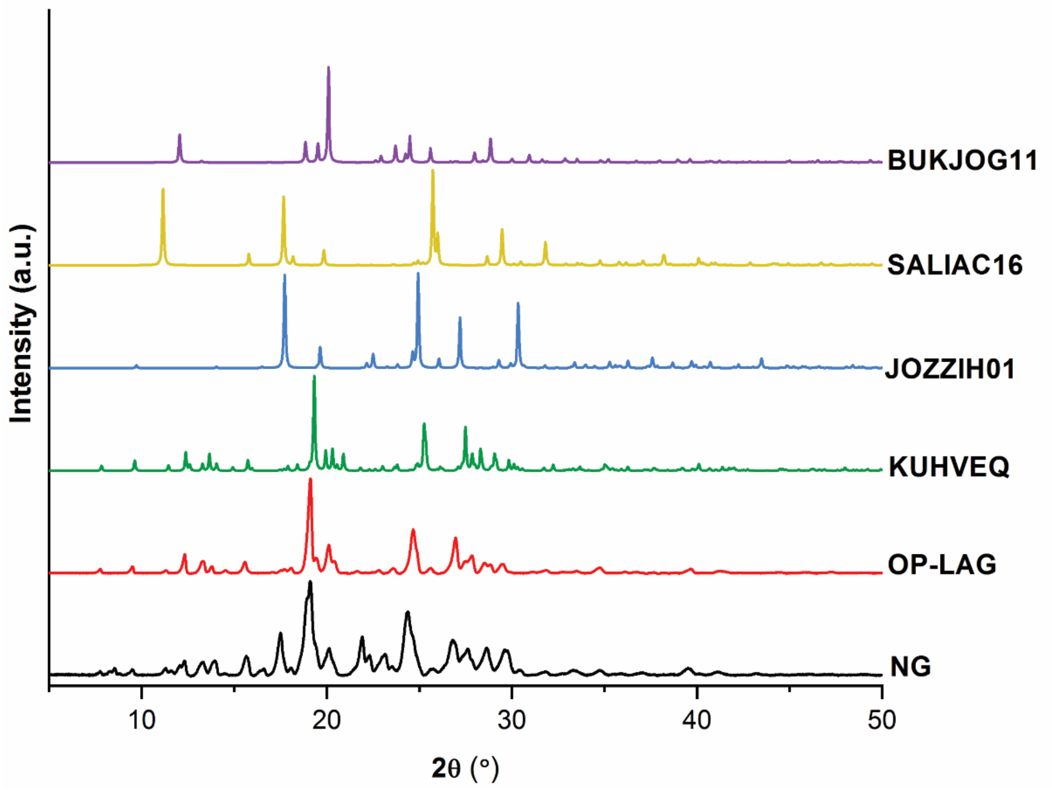


**Figure S4:** Comparison of the experimental PXRD patterns for the neat grinding (NG) and one-pot liquid-assisted grinding (OP-LAG, MeOH) products with the simulated PXRD pattern from the single crystal structure of NCAB-ICC **6** (CSD Refcode: KUHVEQ). JOZZIH01, SALIAC16 and BUKJOG11 are the simulated PXRD patterns from the single crystal structures of 4-hydroxybenzoic acid, 2-hydroxybenzoic acid and 4-dimethylaminopyridine respectively.

## 3. DSC and TGA curves for synthesized solid forms

### 3.1 Figure S5: Combined DSC/TGA curves for 2-CLBZA$^-$·4-DMAPH$^+$ (1) and 2-CLBZA$^-$·4-DMAPH$^+$·1.33$H_2O$ (1a)

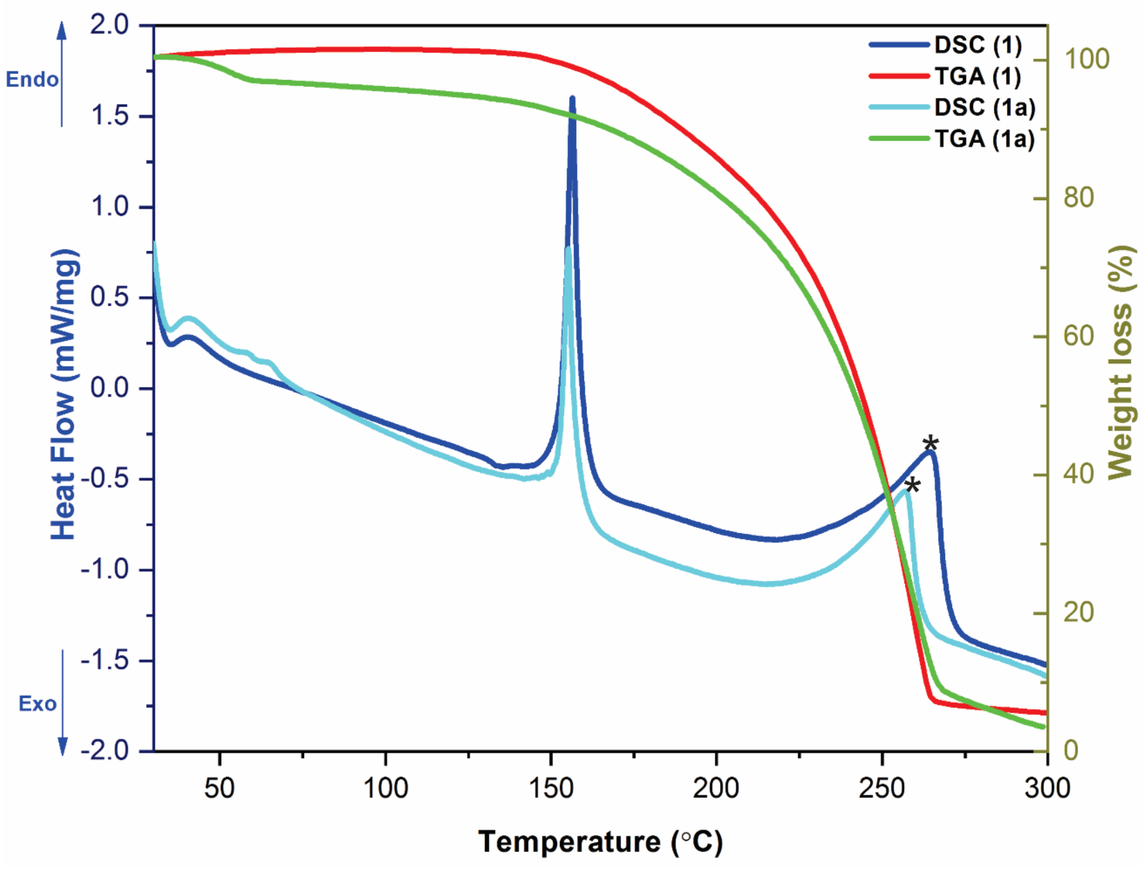


Figure S5: Overlay of the DSC and TGA curves for the 4-dimethylaminopyridine 2-chlorobenzoate (1) salt anhydrate with the comparable data over the same temperature range for the non-stoichiometric hydrate (1a) obtained following LAG experiments with water. Melting is followed by decomposition for both solid forms. * Indicates decomposition events in the DSC curves for 1 and 1a.

3.3 Figure S6: DSC curves for all ICC solid forms (2-7)

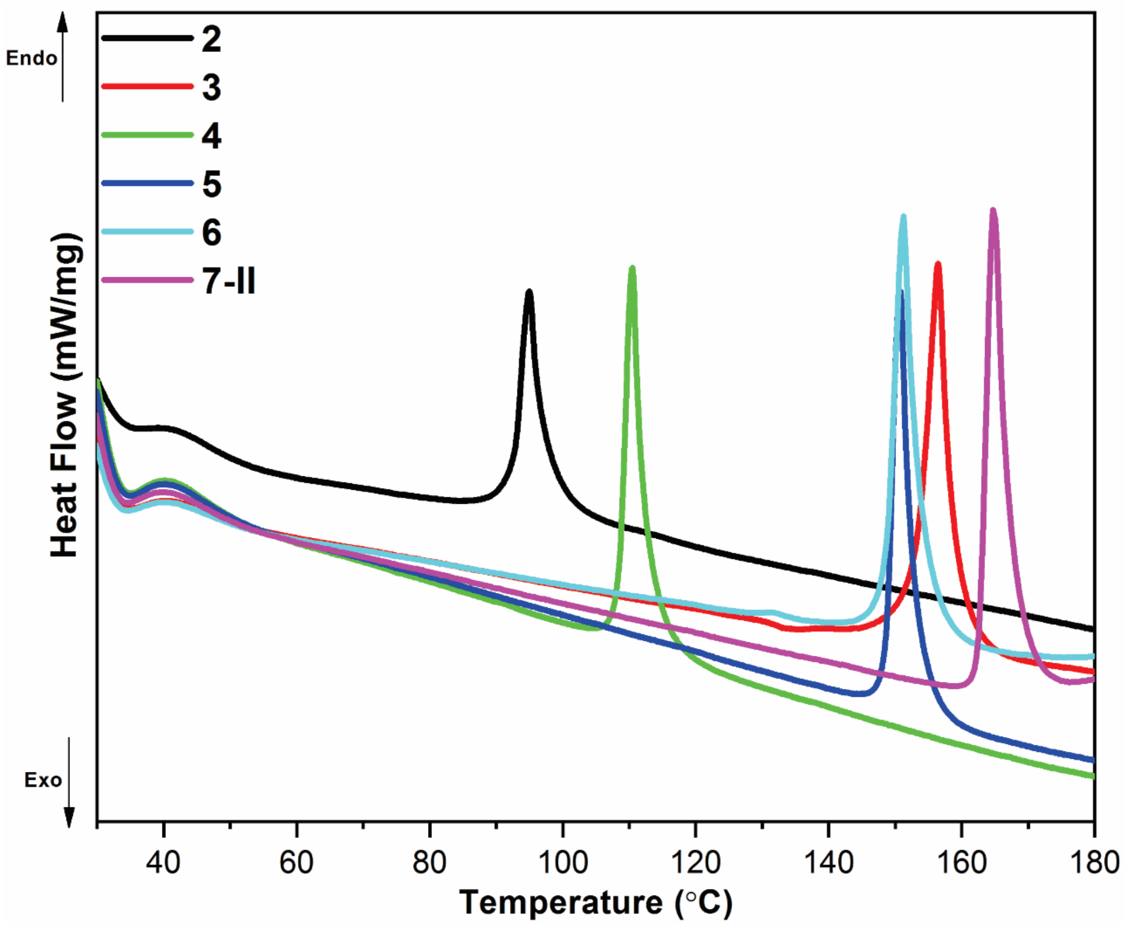


Figure S6: Overlay of the DSC curves for ICCs **2**-**6** and **7-II** indicating the melting endotherms.

# 4. Computational modelling

## 4.1 Table S3: DFT-D estimates of the lattice energies for ICCs and the corresponding binary salt and acid coformers

| ICC Solid Form Number | ICC Composition | Acid Coformer | | Binary Salt | | Ternary ICC | | ICC Stabilization Energy ($\Delta E_{\mathrm{ICC}}$/ $kJ\ mol^{-1}$) |
|---|---|---|---|---|---|---|---|---|
| | | CSD Refcode/Solid Form Number | TPSS+D3 reduced energy / eV | CSD Refcode/Solid Form Number | TPSS+D3 reduced energy / eV | CSD Refcode/Solid Form Number | TPSS+D3 reduced energy / eV | |
| 2 | 2-CLBZA$^-$·4-DMAPH$^+$·2-CLBZAH | CLBZAC03 | -119.54 | 1 | -252.32 | 2 | -371.86 | -0.11 |
| 3 | 4-CLBZA$^-$·4-DMAPH$^+$·4-CLBZAH | CLBZAP03 | -119.77 | CUKNED | -252.45 | CUKNON | -372.27 | -1.45 |
| 4 | 2-HBZA$^-$·4-DMAPH$^+$·2-HBZAH | SALIAC16 | -124.70 | KUJDEA | -257.64 | KUJDIE | -382.36 | -0.69 |
| 5 | 2-HBZA$^-$·4-DMAPH$^+$·4-CLBZAH | CLBZAP03 | -119.77 | KUJDEA | -257.64 | KUHVAM | -377.43 | -0.63 |
| 6 | 2-HBZA$^-$·4-DMAPH$^+$·4-HBZAH | JOZZIH01 | -124.68 | KUJDEA | -257.64 | KUHVEQ | -382.47 | -4.86 |
| 7-I | 4-HBZA$^-$·4-DMAPH$^+$·4-HBZAH | JOZZIH01 | -124.68 | SOLGUX | -257.59 | CUKNUT | -382.33 | -1.86 |
| 7-II | 4-HBZA$^-$·4-DMAPH$^+$·4-HBZAH | JOZZIH01 | -124.68 | SOLGUX | -257.59 | 7-II | -382.41 | -4.42 |

Table S3: TPSS+D3 estimates of the lattice energies for each ICC as well as the comparable values for the binary salt and acid coformer used to synthesize each ICC. The reduced energy is the total TPSS+D3 energy for each solid form scaled by the product of Z and the number of chemically distinct species in the asymmetric unit. The ICC Stabilization Energy is defined in Equation 2 of the manuscript.

## 4.2 Table S4: Selected most stable predicted structures for binary salt 1

| Rank | $\Delta E_{latt}$ / kJ mol$^{-1}$ | Space Group | a (Å) | b (Å) | c (Å) | α (°) | β (°) | γ (°) | Density / g cm$^{-3}$ |
|---|---|---|---|---|---|---|---|---|---|
| 1 | 0.00 | $P2_1/c$ | 10.96 | 10.24 | 15.75 | 90.00 | 126.48 | 90.00 | 1.30 |
| 2 | 0.75 | $P2_1/c$ | 10.07 | 16.53 | 8.56 | 90.00 | 84.68 | 90.00 | 1.31 |
| 3 | 1.41 | Pbca | 10.84 | 30.96 | 8.54 | 90.00 | 90.00 | 90.00 | 1.29 |
| 4 | 1.80 | $P2_1/c$ | 11.09 | 9.89 | 13.17 | 90.00 | 81.94 | 90.00 | 1.30 |
| 5 | 2.02 | $P2_1/c$ | 12.13 | 12.10 | 9.14 | 90.00 | 87.87 | 90.00 | 1.38 |
| 6 | 2.03 | $P2_1/c$ | 9.58 | 11.31 | 15.64 | 90.00 | 55.22 | 90.00 | 1.33 |
| 7 | 2.33 | $P2_1/c$ | 8.64 | 16.47 | 13.73 | 90.00 | 133.35 | 90.00 | 1.30 |
| 8 | 3.15 | P21 | 9.57 | 12.63 | 7.44 | 90.00 | 49.48 | 90.00 | 1.36 |
| 9 | 3.33 | $P2_1/c$ | 12.88 | 11.25 | 9.65 | 90.00 | 93.72 | 90.00 | 1.33 |
| 10 | 3.59 | $P2_1/c$ | 12.28 | 8.13 | 15.21 | 90.00 | 75.44 | 90.00 | 1.26 |
| 11 | 3.88 | $P2_1/c$ | 9.08 | 12.07 | 15.57 | 90.00 | 52.48 | 90.00 | 1.37 |
| 12 | 4.34 | Pbca | 15.33 | 11.69 | 15.37 | 90.00 | 90.00 | 90.00 | 1.34 |
| 13 | 4.41 | $P2_1/c$ | 16.09 | 8.82 | 10.63 | 90.00 | 74.09 | 90.00 | 1.28 |
| 14 | 4.43 | $P2_1/c$ | 9.65 | 11.31 | 15.53 | 90.00 | 55.37 | 90.00 | 1.33 |
| 15 | 4.67 | $P2_1/c$ | 10.08 | 9.28 | 16.11 | 90.00 | 72.06 | 90.00 | 1.29 |
| 16 | 4.79 | P21 | 7.38 | 12.63 | 7.38 | 90.00 | 98.91 | 90.00 | 1.36 |
| 17 | 5.13 | $P2_1/c$ | 8.31 | 10.29 | 17.35 | 90.00 | 74.03 | 90.00 | 1.30 |
| 18 | 5.17 | Cc | 12.22 | 12.56 | 11.64 | 90.00 | 55.43 | 90.00 | 1.26 |
| 19 | 5.36 | $P2_1/c$ | 8.61 | 23.76 | 6.93 | 90.00 | 84.31 | 90.00 | 1.31 |
| 20 | 5.96 | Pbca | 14.89 | 13.55 | 13.83 | 90.00 | 90.00 | 90.00 | 1.33 |
| 21 | 6.00 | C2/c | 30.56 | 8.90 | 10.53 | 90.00 | 87.48 | 90.00 | 1.29 |
| 22 | 6.00 | Pbca | 15.01 | 13.57 | 13.73 | 90.00 | 90.00 | 90.00 | 1.32 |
| 23 | 6.01 | P-1 | 7.45 | 9.77 | 11.77 | 85.45 | 62.16 | 80.62 | 1.24 |
| 24 | 6.27 | Pbca | 15.37 | 15.59 | 11.72 | 90.00 | 90.00 | 90.00 | 1.32 |
| 25 | 6.35 | P21 | 10.91 | 9.89 | 7.36 | 90.00 | 119.81 | 90.00 | 1.34 |
| 26 | 6.39 | $P2_1/c$ | 8.50 | 21.59 | 7.98 | 90.00 | 92.53 | 90.00 | 1.27 |
| 27 | 6.56 | $P2_1/c$ | 11.86 | 7.90 | 17.29 | 90.00 | 115.47 | 90.00 | 1.27 |
| 28 | 6.67 | P-1 | 7.83 | 9.02 | 12.25 | 103.60 | 62.19 | 95.20 | 1.24 |
| 29 | 6.89 | $P2_1/c$ | 15.19 | 12.72 | 7.59 | 90.00 | 87.19 | 90.00 | 1.26 |
| 30 | 6.91 | $P2_1/c$ | 8.71 | 12.50 | 14.39 | 90.00 | 115.91 | 90.00 | 1.31 |

**Table S4:** Selected most stable lattice energy minima for binary salt **1.** The above lattice energy minima were calculated following CrystalOptimizer refinement of the predicted structures using the flexible degrees of freedom defined in Figure 8 of the manuscript. The lattice energy ($\Delta E_{latt}$) for each structure is quoted relative to the lattice energy of the global minimum structure. The experimental structure is indicated in the blue row.

## 4.3 Table S5: Selected most stable predicted structures for CAB-ICC 2

| Rank | $\Delta E_{latt}$ / kJ mol$^{-1}$ | Space Group | a (Å) | b (Å) | c (Å) | α (°) | β (°) | γ (°) | Density / g cm$^{-3}$ |
|---|---|---|---|---|---|---|---|---|---|
| 1 | 0.00 | P-1 | 7.58 | 10.77 | 13.70 | 79.07 | 91.82 | 107.77 | 1.38 |
| 2 | 2.00 | P-1 | 10.89 | 13.56 | 7.60 | 88.29 | 71.59 | 79.68 | 1.38 |
| 3 | 3.32 | P-1 | 13.62 | 7.63 | 11.10 | 68.20 | 99.11 | 88.05 | 1.37 |
| 4 | 3.86 | P-1 | 8.25 | 7.08 | 18.55 | 91.19 | 83.33 | 85.14 | 1.35 |
| 5 | 6.46 | P-1 | 8.23 | 17.56 | 7.26 | 90.20 | 90.58 | 87.91 | 1.38 |
| 6 | 6.55 | P-1 | 7.62 | 20.34 | 7.70 | 101.60 | 85.98 | 112.27 | 1.34 |
| 7 | 7.01 | P-1 | 18.01 | 8.44 | 7.10 | 96.37 | 87.12 | 80.40 | 1.37 |
| 8 | 7.42 | $P2_1$ | 6.96 | 9.98 | 15.24 | 90.00 | 89.59 | 90.00 | 1.36 |
| 9 | 8.19 | P-1 | 15.27 | 7.51 | 10.60 | 70.93 | 96.70 | 72.53 | 1.36 |
| 10 | 8.58 | P-1 | 19.08 | 7.40 | 10.58 | 107.90 | 84.15 | 54.47 | 1.34 |
| 11 | 8.88 | P-1 | 7.11 | 10.71 | 19.53 | 76.62 | 59.67 | 109.06 | 1.36 |
| 12 | 9.18 | P-1 | 6.91 | 21.43 | 17.14 | 58.80 | 61.80 | 112.78 | 1.39 |
| 13 | 9.23 | P-1 | 14.05 | 8.44 | 10.72 | 95.31 | 75.73 | 61.89 | 1.37 |
| 14 | 9.83 | P-1 | 8.55 | 7.35 | 17.63 | 93.76 | 81.68 | 80.02 | 1.35 |
| 15 | 10.03 | P-1 | 13.20 | 10.48 | 8.51 | 93.00 | 89.44 | 66.26 | 1.34 |
| 16 | 10.08 | $P2_1/c$ | 7.10 | 38.07 | 8.02 | 90.00 | 88.32 | 90.00 | 1.33 |
| 17 | 10.20 | P-1 | 7.45 | 14.40 | 11.19 | 97.87 | 66.99 | 83.47 | 1.34 |
| 18 | 10.23 | P-1 | 7.55 | 10.60 | 15.38 | 82.44 | 73.74 | 110.61 | 1.35 |
| 19 | 11.13 | P-1 | 10.49 | 13.16 | 8.55 | 87.99 | 92.59 | 66.19 | 1.34 |
| 20 | 11.14 | P-1 | 18.53 | 8.02 | 7.28 | 94.88 | 91.27 | 86.20 | 1.34 |
| 21 | 11.84 | P-1 | 15.30 | 11.14 | 6.86 | 66.14 | 85.23 | 77.77 | 1.38 |
| 22 | 12.00 | P-1 | 7.20 | 8.31 | 19.34 | 70.33 | 91.18 | 92.85 | 1.33 |
| 23 | 12.09 | P-1 | 7.05 | 9.47 | 17.83 | 95.12 | 76.06 | 69.74 | 1.36 |
| 24 | 12.26 | $P2_1/c$ | 7.45 | 8.41 | 34.25 | 90.00 | 90.16 | 90.00 | 1.35 |
| 25 | 12.27 | $P2_12_12_1$ | 14.74 | 18.86 | 7.67 | 90.00 | 90.00 | 90.00 | 1.36 |
| 26 | 12.50 | P-1 | 7.19 | 10.39 | 19.02 | 75.54 | 57.76 | 103.09 | 1.38 |
| 27 | 12.55 | $P2_1/c$ | 14.74 | 10.79 | 14.38 | 90.00 | 110.80 | 90.00 | 1.35 |
| 28 | 12.56 | $P2_1/c$ | 7.29 | 16.92 | 19.33 | 90.00 | 117.75 | 90.00 | 1.37 |
| 29 | 12.65 | P-1 | 8.70 | 7.37 | 17.23 | 89.57 | 80.48 | 92.04 | 1.33 |
| 30 | 12.67 | $P2_1$ | 9.30 | 17.50 | 7.71 | 90.00 | 58.19 | 90.00 | 1.36 |

**Table S5:** Selected most stable lattice energy minima for CAB-ICC **2.** The above lattice energy minima were calculated following CrystalOptimizer refinement of the predicted structures using the flexible degrees of freedom defined in Figure 8 of the manuscript. The lattice energy ($\Delta E_{latt}$) for each structure is quoted relative to the lattice energy of the global minimum structure. The experimental structure is indicated in the blue row.

## 4.4 Table S6: Selected most stable predicted structures for CAB-ICC 3

| Rank | $\Delta E_{latt}$ / kJ mol$^{-1}$ | Space Group | a (Å) | b (Å) | c (Å) | α (°) | β (°) | γ (°) | Density / g cm$^{-3}$ |
|---|---|---|---|---|---|---|---|---|---|
| 1 | 0.00 | P-1 | 13.18 | 10.02 | 9.93 | 58.76 | 86.95 | 104.08 | 1.36 |
| 2 | 3.43 | P-1 | 10.00 | 9.94 | 12.68 | 77.07 | 86.18 | 63.65 | 1.31 |
| 3 | 3.44 | P-1 | 9.94 | 12.68 | 10.00 | 86.18 | 63.64 | 77.06 | 1.32 |
| 4 | 4.61 | $P2_1/c$ | 19.13 | 7.53 | 16.89 | 90.00 | 63.35 | 90.00 | 1.33 |
| 5 | 4.63 | $P2_1/c$ | 19.13 | 7.53 | 16.89 | 90.00 | 63.34 | 90.00 | 1.33 |
| 6 | 4.80 | $P2_1/c$ | 15.57 | 7.37 | 19.49 | 90.00 | 73.39 | 90.00 | 1.35 |
| 7 | 4.80 | $P2_1/c$ | 15.57 | 7.37 | 19.49 | 90.00 | 73.39 | 90.00 | 1.35 |
| 8 | 5.63 | P-1 | 7.52 | 15.95 | 9.51 | 76.95 | 98.77 | 98.75 | 1.33 |
| 9 | 6.09 | $P2_1/c$ | 15.51 | 9.83 | 16.92 | 90.00 | 57.73 | 90.00 | 1.33 |
| 10 | 6.45 | P-1 | 13.40 | 9.55 | 9.87 | 61.62 | 89.17 | 76.34 | 1.35 |
| 11 | 6.49 | $P2_1/c$ | 10.29 | 10.64 | 20.25 | 90.00 | 75.69 | 90.00 | 1.35 |
| 12 | 6.50 | $P2_1/c$ | 10.29 | 10.64 | 20.25 | 90.00 | 104.31 | 90.00 | 1.35 |
| 13 | 6.53 | $P2_1/c$ | 12.17 | 9.70 | 18.51 | 90.00 | 78.83 | 90.00 | 1.35 |
| 14 | 6.54 | P-1 | 9.19 | 9.21 | 14.36 | 88.14 | 101.58 | 65.13 | 1.35 |
| 15 | 6.55 | P-1 | 14.36 | 9.19 | 9.21 | 65.14 | 91.86 | 78.43 | 1.35 |
| 16 | 6.62 | $P2_1/c$ | 9.48 | 7.53 | 31.48 | 90.00 | 98.92 | 90.00 | 1.30 |
| 17 | 6.66 | $P2_1/c$ | 7.43 | 18.35 | 16.40 | 90.00 | 77.22 | 90.00 | 1.33 |
| 18 | 6.67 | $P2_1/c$ | 7.43 | 18.35 | 16.40 | 90.00 | 77.22 | 90.00 | 1.33 |
| 19 | 6.83 | P-1 | 9.08 | 14.13 | 9.69 | 81.32 | 61.86 | 94.26 | 1.35 |
| 20 | 6.90 | $P2_1/c$ | 14.43 | 9.79 | 15.38 | 90.00 | 94.68 | 90.00 | 1.34 |
| 21 | 6.90 | $P2_1/c$ | 14.43 | 9.78 | 15.38 | 90.00 | 94.69 | 90.00 | 1.34 |
| 22 | 7.28 | $P2_1/c$ | 7.72 | 14.08 | 19.87 | 90.00 | 90.75 | 90.00 | 1.34 |
| 23 | 7.35 | P-1 | 8.74 | 13.77 | 9.24 | 86.38 | 77.14 | 79.07 | 1.36 |
| 24 | 7.39 | $P2_1/c$ | 9.88 | 30.38 | 7.45 | 90.00 | 70.18 | 90.00 | 1.38 |
| 25 | 7.42 | $P2_1/c$ | 9.88 | 30.37 | 7.45 | 90.00 | 70.17 | 90.00 | 1.38 |
| 26 | 7.57 | $P2_1/c$ | 6.95 | 18.12 | 16.79 | 90.00 | 82.56 | 90.00 | 1.38 |
| 27 | 7.58 | C2/c | 9.70 | 16.97 | 27.16 | 90.00 | 90.04 | 90.00 | 1.29 |
| 28 | 7.58 | C2/c | 27.16 | 16.97 | 9.70 | 90.00 | 90.03 | 90.00 | 1.29 |
| 29 | 7.59 | C2/c | 16.26 | 9.75 | 27.35 | 90.00 | 80.77 | 90.00 | 1.35 |
| 30 | 7.65 | $P2_12_12_1$ | 33.46 | 7.57 | 8.30 | 90.00 | 90.00 | 90.00 | 1.37 |

**Table S6:** Selected most stable rigid body lattice energy minima for CAB-ICC **3**. The lattice energy ($\Delta E_{latt}$) for each structure is quoted relative to the lattice energy of the global minimum structure. The experimental structure is indicated in the blue row.

## 4.5 Table S7: Selected most stable predicted structures for CAB-ICC 4

| Rank | $\Delta E_{latt}$ / kJ mol$^{-1}$ | Space Group | a (Å) | b (Å) | c (Å) | α (°) | β (°) | γ (°) | Density / g cm$^{-3}$ |
|---|---|---|---|---|---|---|---|---|---|
| 1 | 0.00 | $P2_1/c$ | 9.94 | 13.48 | 15.42 | 90.00 | 77.97 | 90.00 | 1.31 |
| 2 | 2.46 | $P2_12_12_1$ | 10.77 | 18.17 | 10.23 | 90.00 | 90.00 | 90.00 | 1.32 |
| 3 | 2.84 | P-1 | 7.88 | 16.58 | 8.38 | 93.80 | 73.30 | 83.76 | 1.28 |
| 4 | 2.95 | P-1 | 8.81 | 9.57 | 13.13 | 93.40 | 90.57 | 65.51 | 1.32 |
| 5 | 3.47 | $P2_1/c$ | 9.71 | 13.15 | 17.50 | 90.00 | 115.15 | 90.00 | 1.31 |
| 6 | 3.51 | $P2_1/c$ | 19.62 | 12.89 | 8.37 | 90.00 | 109.54 | 90.00 | 1.33 |
| 7 | 4.39 | $P2_12_12_1$ | 9.80 | 15.54 | 13.47 | 90.00 | 90.00 | 90.00 | 1.29 |
| 8 | 4.50 | $P2_1/c$ | 15.80 | 9.58 | 13.20 | 90.00 | 87.88 | 90.00 | 1.32 |
| 9 | 4.64 | P-1 | 7.44 | 17.75 | 9.23 | 105.79 | 68.72 | 77.99 | 1.29 |
| 10 | 4.69 | $P2_12_12_1$ | 9.87 | 15.30 | 13.51 | 90.00 | 90.00 | 90.00 | 1.30 |
| 11 | 4.89 | $P2_1/c$ | 7.67 | 21.63 | 14.54 | 90.00 | 124.60 | 90.00 | 1.33 |
| 12 | 4.89 | $P2_1/c$ | 11.99 | 21.63 | 7.67 | 90.00 | 87.19 | 90.00 | 1.33 |
| 13 | 5.03 | $P2_1/c$ | 13.72 | 9.79 | 15.08 | 90.00 | 88.67 | 90.00 | 1.31 |
| 14 | 5.45 | $P2_1/c$ | 13.22 | 15.72 | 10.55 | 90.00 | 67.54 | 90.00 | 1.31 |
| 15 | 5.81 | $P2_1/c$ | 7.77 | 9.81 | 31.12 | 90.00 | 59.23 | 90.00 | 1.30 |
| 16 | 5.81 | $P2_1/c$ | 26.74 | 9.81 | 7.77 | 90.00 | 90.83 | 90.00 | 1.30 |
| 17 | 6.00 | $P2_1/c$ | 9.76 | 13.75 | 15.50 | 90.00 | 80.87 | 90.00 | 1.29 |
| 18 | 6.02 | $P2_1/c$ | 9.76 | 13.75 | 15.51 | 90.00 | 80.87 | 90.00 | 1.29 |
| 19 | 6.35 | $P2_1/c$ | 13.87 | 9.81 | 14.95 | 90.00 | 88.27 | 90.00 | 1.30 |
| 20 | 6.40 | $P2_1/c$ | 16.19 | 9.72 | 15.05 | 90.00 | 120.43 | 90.00 | 1.30 |
| 21 | 6.49 | $P2_1/c$ | 10.19 | 15.76 | 13.34 | 90.00 | 110.15 | 90.00 | 1.32 |
| 22 | 6.50 | P-1 | 12.51 | 7.09 | 12.90 | 87.81 | 63.44 | 83.45 | 1.30 |
| 23 | 6.52 | $P2_1/c$ | 9.96 | 15.52 | 13.68 | 90.00 | 72.77 | 90.00 | 1.31 |
| 24 | 6.53 | $P2_1/c$ | 9.96 | 15.52 | 13.68 | 90.00 | 72.77 | 90.00 | 1.31 |
| 25 | 6.71 | $P2_1/c$ | 9.77 | 15.59 | 13.49 | 90.00 | 86.18 | 90.00 | 1.29 |
| 26 | 6.72 | $P2_1/c$ | 9.78 | 15.59 | 13.49 | 90.00 | 86.14 | 90.00 | 1.29 |
| 27 | 6.90 | $P2_1/c$ | 15.77 | 13.18 | 9.87 | 90.00 | 83.92 | 90.00 | 1.30 |
| 28 | 7.33 | $P2_1/c$ | 9.22 | 23.09 | 10.70 | 90.00 | 63.21 | 90.00 | 1.30 |
| 29 | 7.34 | $P2_1/c$ | 10.70 | 23.09 | 9.22 | 90.00 | 63.21 | 90.00 | 1.30 |
| 30 | 7.35 | $Pna2_1$ | 13.50 | 15.61 | 9.70 | 90.00 | 90.00 | 90.00 | 1.29 |

**Table S7:** Selected most stable rigid body lattice energy minima for CAB-ICC **4.** The lattice energy ($\Delta E_{latt}$) for each structure is quoted relative to the lattice energy of the global minimum structure. The experimental structure is indicated in the blue row.

## 4.6 Table S8: Selected most stable predicted structures for NCAB-ICC 5

| Rank | $\Delta E_{latt}$ / kJ mol$^{-1}$ | Space Group | a (Å) | b (Å) | c (Å) | α (°) | β (°) | γ (°) | Density / g cm$^{-3}$ |
|---|---|---|---|---|---|---|---|---|---|
| 1 | 0.00 | P-1 | 9.68 | 9.80 | 16.33 | 91.92 | 53.15 | 117.67 | 1.34 |
| 2 | 3.58 | P-1 | 14.95 | 9.71 | 9.06 | 61.55 | 97.50 | 71.39 | 1.34 |
| 3 | 4.53 | P-1 | 9.51 | 15.06 | 9.06 | 105.83 | 62.56 | 79.56 | 1.34 |
| 4 | 4.60 | $P2_1/c$ | 7.58 | 28.68 | 10.19 | 90.00 | 66.69 | 90.00 | 1.36 |
| 5 | 4.72 | P-1 | 9.07 | 7.53 | 16.92 | 83.92 | 92.02 | 67.94 | 1.30 |
| 6 | 5.05 | P-1 | 9.52 | 8.38 | 17.99 | 103.54 | 81.82 | 129.33 | 1.28 |
| 7 | 5.23 | P-1 | 9.68 | 9.78 | 12.84 | 94.16 | 81.26 | 62.43 | 1.32 |
| 8 | 5.49 | $P2_1/c$ | 14.35 | 7.45 | 19.49 | 90.00 | 99.35 | 90.00 | 1.35 |
| 9 | 5.50 | $P2_1/c$ | 21.18 | 7.45 | 12.88 | 90.00 | 88.82 | 90.00 | 1.36 |
| 10 | 6.17 | P-1 | 11.72 | 16.28 | 7.52 | 60.43 | 55.42 | 75.37 | 1.35 |
| 11 | 6.20 | P-1 | 9.88 | 12.95 | 9.49 | 88.75 | 63.37 | 106.31 | 1.35 |
| 12 | 6.27 | P-1 | 13.82 | 8.96 | 9.63 | 62.38 | 100.40 | 87.39 | 1.35 |
| 13 | 7.00 | C2/c | 42.16 | 7.87 | 12.78 | 90.00 | 103.04 | 90.00 | 1.34 |
| 14 | 7.00 | $P2_1/c$ | 9.60 | 27.85 | 9.49 | 90.00 | 56.61 | 90.00 | 1.31 |
| 15 | 7.14 | C2/c | 26.65 | 9.74 | 16.16 | 90.00 | 84.39 | 90.00 | 1.33 |
| 16 | 7.24 | C2/c | 16.76 | 9.86 | 26.05 | 90.00 | 87.73 | 90.00 | 1.29 |
| 17 | 7.37 | $P2_1/c$ | 24.24 | 7.41 | 12.56 | 90.00 | 65.77 | 90.00 | 1.35 |
| 18 | 7.48 | $P2_1/c$ | 15.98 | 9.76 | 14.82 | 90.00 | 62.11 | 90.00 | 1.35 |
| 19 | 7.61 | $P2_1/c$ | 16.84 | 13.10 | 9.68 | 90.00 | 82.45 | 90.00 | 1.31 |
| 20 | 7.68 | C2/c | 17.11 | 7.46 | 33.14 | 90.00 | 94.02 | 90.00 | 1.31 |
| 21 | 7.88 | $P2_1/c$ | 13.98 | 16.16 | 9.60 | 90.00 | 70.91 | 90.00 | 1.35 |
| 22 | 7.91 | C2/c | 27.01 | 9.68 | 15.97 | 90.00 | 86.18 | 90.00 | 1.33 |
| 23 | 8.14 | P-1 | 12.65 | 9.71 | 8.69 | 93.14 | 81.47 | 93.56 | 1.32 |
| 24 | 8.24 | $P2_1/c$ | 8.85 | 27.43 | 9.59 | 90.00 | 62.86 | 90.00 | 1.34 |
| 25 | 8.31 | $P2_1/c$ | 14.17 | 9.78 | 14.77 | 90.00 | 89.16 | 90.00 | 1.35 |
| 26 | 8.38 | C2/c | 15.90 | 9.72 | 27.08 | 90.00 | 85.31 | 90.00 | 1.33 |
| 27 | 8.51 | P-1 | 8.99 | 13.69 | 9.63 | 75.84 | 63.36 | 78.14 | 1.36 |
| 28 | 8.51 | $P2_1/c$ | 8.92 | 33.05 | 7.72 | 90.00 | 71.14 | 90.00 | 1.29 |
| 29 | 8.53 | $P2_1/c$ | 7.68 | 14.35 | 19.64 | 90.00 | 71.04 | 90.00 | 1.35 |
| 30 | 8.59 | $P2_1/c$ | 13.61 | 16.37 | 9.90 | 90.00 | 70.78 | 90.00 | 1.33 |

**Table S8:** Selected most stable rigid body lattice energy minima for NCAB-ICC **5**. The lattice energy ($\Delta E_{latt}$) for each structure is quoted relative to the lattice energy of the global minimum structure. The experimental structure is indicated in the blue row.

## 4.7 Table S9: Selected most stable predicted structures for NCAB-ICC 6

| Rank | $\Delta E_{latt}$ / kJ mol$^{-1}$ | Space Group | a (Å) | b (Å) | c (Å) | α (°) | β (°) | γ (°) | Density / g cm$^{-3}$ |
|---|---|---|---|---|---|---|---|---|---|
| 1 | 0.00 | $P2_1/c$ | 27.00 | 7.77 | 9.87 | 90.00 | 84.49 | 90.00 | 1.28 |
| 2 | 0.40 | $P2_1/c$ | 15.37 | 7.94 | 19.43 | 90.00 | 59.07 | 90.00 | 1.30 |
| 3 | 1.94 | $P2_1/c$ | 15.48 | 9.73 | 14.37 | 90.00 | 111.72 | 90.00 | 1.32 |
| 4 | 2.08 | $P2_1/c$ | 14.40 | 9.83 | 14.42 | 90.00 | 84.54 | 90.00 | 1.30 |
| 5 | 3.21 | Pbcn | 21.93 | 14.58 | 13.08 | 90.00 | 90.00 | 90.00 | 1.27 |
| 6 | 3.63 | $P2_12_12_1$ | 10.22 | 26.84 | 7.21 | 90.00 | 90.00 | 90.00 | 1.34 |
| 7 | 3.84 | Pbca | 14.86 | 27.95 | 10.09 | 90.00 | 90.00 | 90.00 | 1.26 |
| 8 | 4.00 | $Pna2_1$ | 22.85 | 12.58 | 7.33 | 90.00 | 90.00 | 90.00 | 1.26 |
| 9 | 4.16 | C2/c | 19.62 | 7.87 | 30.49 | 90.00 | 60.52 | 90.00 | 1.29 |
| 10 | 4.24 | $P2_1/c$ | 14.77 | 10.29 | 15.23 | 90.00 | 63.26 | 90.00 | 1.28 |
| 11 | 4.25 | C2/c | 17.86 | 8.39 | 27.56 | 90.00 | 95.99 | 90.00 | 1.29 |
| 12 | 4.27 | $P2_1/c$ | 7.35 | 19.04 | 14.14 | 90.00 | 88.45 | 90.00 | 1.34 |
| 13 | 4.27 | $P2_1/c$ | 7.20 | 10.19 | 30.04 | 90.00 | 116.24 | 90.00 | 1.34 |
| 14 | 4.45 | $P2_1/c$ | 9.83 | 7.32 | 28.50 | 90.00 | 85.82 | 90.00 | 1.29 |
| 15 | 4.58 | C2/c | 19.46 | 7.48 | 28.44 | 90.00 | 89.39 | 90.00 | 1.28 |
| 16 | 4.81 | $P2_1/c$ | 9.81 | 27.35 | 7.73 | 90.00 | 93.37 | 90.00 | 1.28 |
| 17 | 5.22 | $P2_1/c$ | 14.68 | 22.91 | 7.21 | 90.00 | 59.66 | 90.00 | 1.27 |
| 18 | 5.25 | $P2_1/c$ | 8.15 | 26.14 | 9.84 | 90.00 | 95.61 | 90.00 | 1.27 |
| 19 | 5.30 | $P2_1$ | 12.57 | 7.32 | 13.00 | 90.00 | 61.76 | 90.00 | 1.26 |
| 20 | 5.33 | P-1 | 9.59 | 6.91 | 15.78 | 96.55 | 82.79 | 77.01 | 1.32 |
| 21 | 5.35 | $P2_1/c$ | 7.64 | 14.33 | 19.69 | 90.00 | 111.96 | 90.00 | 1.32 |
| 22 | 5.84 | Pbcn | 22.08 | 14.49 | 13.18 | 90.00 | 90.00 | 90.00 | 1.26 |
| 23 | 5.87 | $P2_1/c$ | 16.68 | 6.97 | 18.76 | 90.00 | 66.11 | 90.00 | 1.33 |
| 24 | 5.97 | $P2_1/c$ | 16.71 | 13.98 | 8.76 | 90.00 | 79.98 | 90.00 | 1.31 |
| 25 | 6.00 | $P2_12_12_1$ | 12.60 | 7.29 | 22.94 | 90.00 | 90.00 | 90.00 | 1.26 |
| 26 | 6.05 | $Pna2_1$ | 7.26 | 22.94 | 12.63 | 90.00 | 90.00 | 90.00 | 1.26 |
| 27 | 6.13 | $P2_1/c$ | 9.71 | 30.88 | 6.98 | 90.00 | 74.77 | 90.00 | 1.31 |
| 28 | 6.32 | P-1 | 15.84 | 9.96 | 6.96 | 68.19 | 100.45 | 103.69 | 1.34 |
| 29 | 6.38 | $P2_1/c$ | 9.96 | 11.99 | 19.51 | 90.00 | 60.56 | 90.00 | 1.30 |
| 30 | 6.41 | C2/c | 13.21 | 14.44 | 22.71 | 90.00 | 75.89 | 90.00 | 1.26 |

**Table S9:** Selected most stable rigid body lattice energy minima for NCAB-ICC **6.** The lattice energy ($\Delta E_{latt}$) for each structure is quoted relative to the lattice energy of the global minimum structure. The experimental structure is indicated in the blue row.

## 4.8 Table S10: Selected most stable predicted structures for CAB-ICC 7

| Rank | $\Delta E_{latt}$ / kJ mol$^{-1}$ | Space Group | a (Å) | b (Å) | c (Å) | α (°) | β (°) | γ (°) | Density / g cm$^{-3}$ |
|---|---|---|---|---|---|---|---|---|---|
| 1 | 0.00 | $P2_1/c$ | 9.69 | 18.80 | 11.74 | 90.00 | 72.98 | 90.00 | 1.30 |
| 2 | 1.12 | $P2_1/c$ | 14.72 | 10.30 | 15.39 | 90.00 | 63.60 | 90.00 | 1.27 |
| 3 | 1.35 | $P2_1$ | 13.38 | 10.24 | 7.52 | 90.00 | 86.22 | 90.00 | 1.29 |
| 4 | 2.84 | $P2_1/c$ | 7.41 | 18.48 | 15.14 | 90.00 | 79.47 | 90.00 | 1.30 |
| 5 | 3.19 | $P2_1/c$ | 10.26 | 10.11 | 21.44 | 90.00 | 67.39 | 90.00 | 1.29 |
| 6 | 5.08 | $P2_1/c$ | 7.84 | 10.22 | 26.25 | 90.00 | 82.39 | 90.00 | 1.27 |
| 7 | 6.52 | $P2_1/c$ | 7.37 | 14.46 | 24.65 | 90.00 | 124.34 | 90.00 | 1.22 |
| 8 | 6.95 | $P2_1/c$ | 7.41 | 18.60 | 16.19 | 90.00 | 63.29 | 90.00 | 1.33 |
| 9 | 7.30 | $P2_1/c$ | 13.92 | 10.27 | 14.84 | 90.00 | 91.71 | 90.00 | 1.25 |
| 10 | 8.52 | C2/c | 22.97 | 8.12 | 25.40 | 90.00 | 118.18 | 90.00 | 1.27 |
| 11 | 8.98 | $Pna2_1$ | 27.71 | 7.32 | 10.25 | 90.00 | 90.00 | 90.00 | 1.27 |
| 12 | 9.06 | Pbca | 17.89 | 18.57 | 12.54 | 90.00 | 90.00 | 90.00 | 1.27 |
| 13 | 9.15 | P-1 | 7.54 | 13.85 | 10.39 | 94.57 | 89.02 | 95.59 | 1.23 |
| 14 | 9.21 | $P2_1/c$ | 11.43 | 12.19 | 16.55 | 90.00 | 68.21 | 90.00 | 1.24 |
| 15 | 9.69 | $P2_1/c$ | 7.43 | 14.73 | 19.58 | 90.00 | 68.49 | 90.00 | 1.33 |
| 16 | 9.78 | C2/c | 25.22 | 8.82 | 18.64 | 90.00 | 83.24 | 90.00 | 1.29 |
| 17 | 9.85 | $P2_1/c$ | 11.26 | 12.35 | 15.45 | 90.00 | 80.03 | 90.00 | 1.25 |
| 18 | 10.13 | P-1 | 8.23 | 11.46 | 12.93 | 64.57 | 91.74 | 107.23 | 1.27 |
| 19 | 10.49 | P-1 | 13.05 | 12.21 | 8.22 | 112.59 | 86.33 | 64.93 | 1.25 |
| 20 | 10.63 | P-1 | 13.64 | 7.72 | 12.45 | 61.87 | 79.95 | 65.43 | 1.26 |
| 21 | 10.67 | Pbca | 17.46 | 20.70 | 11.35 | 90.00 | 90.00 | 90.00 | 1.29 |
| 22 | 10.68 | $P2_1/n$ | 7.65 | 20.40 | 13.84 | 90.00 | 83.05 | 90.00 | 1.23 |
| 23 | 10.69 | Pbca | 17.46 | 20.70 | 11.35 | 90.00 | 90.00 | 90.00 | 1.29 |
| 24 | 10.82 | $P2_1/c$ | 12.75 | 13.80 | 13.38 | 90.00 | 123.17 | 90.00 | 1.34 |
| 25 | 10.82 | C2/c | 20.65 | 9.45 | 24.43 | 90.00 | 121.60 | 90.00 | 1.30 |
| 26 | 10.87 | $P2_1/c$ | 8.06 | 12.85 | 22.33 | 90.00 | 63.60 | 90.00 | 1.28 |
| 27 | 10.99 | Pbca | 9.52 | 18.77 | 22.55 | 90.00 | 90.00 | 90.00 | 1.31 |
| 28 | 11.47 | $P2_1$ | 7.23 | 9.48 | 15.63 | 90.00 | 77.71 | 90.00 | 1.26 |
| 29 | 11.58 | $P2_1/n$ | 7.50 | 27.44 | 10.23 | 90.00 | 82.57 | 90.00 | 1.27 |
| 30 | 11.80 | $P2_1/c$ | 15.99 | 10.25 | 15.98 | 90.00 | 53.28 | 90.00 | 1.26 |

**Table S10:** Selected most stable rigid body lattice energy minima for CAB-ICC **7**. The lattice energy ($\Delta E_{latt}$) for each structure is quoted relative to the lattice energy of the global minimum structure. The experimental CUKNUT (Form I) structure for CAB-ICC **7** is indicated in the blue row.
